# Evaluating a WeChat-Based Intervention to Enhance Influenza Vaccination Knowledge, Attitude, and Behavior Among Chinese University Students Residing in the United Kingdom: Controlled, Quasi-Experimental, Mixed Methods Study

**DOI:** 10.2196/55706

**Published:** 2024-10-24

**Authors:** Lan Li, Caroline E Wood, Patty Kostkova

**Affiliations:** 1 Centre for Digital Public Health in Emergencies, Department for Risk and Disaster Reduction University College London London United Kingdom

**Keywords:** influenza vaccination, intervention study, social media, students, health promotion, mixed methods

## Abstract

**Background:**

University students, who often live in close quarters and engage in frequent social interaction, face a heightened risk of influenza morbidity. Still, vaccination rates among this group, particularly Chinese students, remain consistently low due to limited awareness and insufficient access to vaccinations.

**Objective:**

This study examines the effectiveness of a cocreated WeChat-based intervention that targets mainland Chinese university students in the United Kingdom, aiming to improve their knowledge, attitude, and behavior (KAB) toward seasonal influenza vaccination.

**Methods:**

A quasi-experimental mixed methods design was used, incorporating an intervention and comparison group, with baseline and follow-up self-reported surveys. The study was conducted from December 19, 2022, to January 16, 2023. The primary outcome is the KAB score, which was measured before and after the intervention phases. System-recorded data and user feedback were included in the analysis as secondary outcomes. A series of hypothesis testing methods were applied to test the primary outcomes, and path analysis was used to explore the relationships.

**Results:**

Our study included 596 students, of which 303 (50.8%) were in the intervention group and 293 (49.2%) were in the control group. The intervention group showed significant improvements in knowledge, attitude, and intended behavior scores over time, whereas the control group had only a slight increase in intended behavior scores. When comparing changes between the 2 groups, the intervention group displayed significant differences in knowledge and attitude scores compared to the control group, while intended behavior scores did not significantly differ. After the intervention, the actual vaccination rate was slightly higher in the intervention group (63/303, 20.8%) compared to the control group (54/293, 18.4%). Path analysis found that the intervention had a significant direct impact on knowledge but not on attitudes; knowledge strongly influenced attitudes, and both knowledge and attitudes significantly influenced intended behavior; and there was a strong correlation between intended and actual behavior. In the intervention group, participants expressed a high level of satisfaction and positive review of the content and its use.

**Conclusions:**

This study demonstrates how a WeChat intervention effectively improves KAB related to seasonal influenza vaccination among Chinese students, highlighting the potential of social media interventions to drive vaccination behavior change. It contributes to the broader research on digital health intervention effectiveness and lays the groundwork for tailoring similar interventions to different health contexts and populations.

## Introduction

Seasonal influenza vaccination (SIV) is a critical public health intervention aimed at reducing the incidence of influenza and its associated complications. Influenza poses a significant burden on population health, leading to increased morbidity, mortality, and substantial health care costs [1]. In the 2022 to 2023 influenza season, the United Kingdom experienced its highest influenza-related excess deaths since 2017 to 2018, with nearly 15,000 reported in the 2022 to 2023 influenza season [2]. Increasing SIV uptake can lead to significant public health benefits, including reduced strain on health care systems during influenza seasons, lower hospitalization rates, and decreased overall health care expenditures [3]. Effective vaccination campaigns can also prevent the spread of the virus within communities, contributing to herd immunity and protecting populations considered vulnerable, including older adults, young children, and individuals with chronic health conditions [3].

Despite these benefits, SIV vaccination rates remain suboptimal in many populations. University students, while generally at a lower risk for severe influenza complications, are still vulnerable to the impact of the virus. This can lead to reduced academic performance, social disruptions, and prolonged symptoms [4,5]. This vulnerability is particularly pronounced among international students, who may face additional challenges such as adapting to a new environment and navigating an unfamiliar health care system [4-7]. However, vaccination rates among university students, especially Chinese students, remain consistently low due to factors such as limited awareness and insufficient access to vaccinations [4-6,8,9]. Extensive research has been conducted on the factors influencing the attitudes and intentions of university students regarding SIV [4,6,10,11]. These studies have identified a significant gap between intention and actual behavior, with only 30% to 40% of students who intend to get vaccinated following through [12]. Given this discrepancy, the focus should shift from merely identifying the reasons behind low vaccination rates to developing and implementing effective interventions that specifically target this population.

Social media holds significant promise for promoting SIV among university students due to its ubiquitous presence in their daily lives, providing a convenient and accessible platform for disseminating vaccination information [13-16]. However, existing interventions have primarily focused on on-campus programs, often neglecting off-campus influences that play a crucial role in vaccination decisions [8]. Thus, there is a critical need to assess and harness the potential of social media for influencing SIV behavior change. In addition, most studies on social media interventions for SIV have been geographically limited, primarily centered in the United States, highlighting the necessity for a broader global perspective [8,12,17,18]. In addition, the prevailing “one-size-fits-all” approach in intervention design has resulted in only moderate effectiveness, emphasizing the need for tailored interventions that cater to the specific needs of different populations [8,13,14]. Reviews have consistently highlighted the importance of customizing interventions to address the unique characteristics and needs of specific target groups to enhance their effectiveness [8,14].

China is the largest source of international students in the United Kingdom, accounting for >22% of the international student population in 2022 [19]. Chinese students often face language barriers, cultural differences, and limited social interactions [20], which can hinder their access to health care resources [21]. Given that WeChat is the predominant platform among Chinese students, it presents a unique opportunity to deliver targeted health interventions [22,23]. While prior research has explored the potential of WeChat for health interventions, the focus has predominantly revolved around areas such as physical activity [24,25], mental health [24,26], and the management of cancer and chronic diseases [27,28]. However, there is a significant research gap in evaluating the effectiveness of WeChat for promoting vaccination, especially among Chinese university students. Our study aims to address this gap by implementing and evaluating a cocreated WeChat-based intervention specifically targeted at improving knowledge, attitude, and behavior (KAB) regarding SIV. To achieve this, we use a quasi-experimental design to assess the efficacy of the intervention among mainland Chinese university students in the United Kingdom.

## Methods

### Study Design

A quasi-experimental, mixed methods design was used, including an intervention and comparison group with baseline and follow-up assessment. This study was conducted in the United Kingdom, with the intervention taking place from December 19, 2022, to January 16, 2023. The timing was deliberately chosen to engage with students who were uncertain about getting vaccinated, assuming that most vaccine acceptors had already been vaccinated at the beginning of the influenza season (October to November). Notably, during this period, the United Kingdom witnessed its most severe influenza season in the past 5 years [2], and China was grappling with a challenging COVID-19 situation, marked by policy changes related to public health measures [29]. These circumstances heightened the population’s interest in health-related information on social media. To ensure that any observed effects could be attributed to the intervention and not influenced by unrelated factors, a control group was thoughtfully included for comparative effectiveness assessment.

### Intervention Details

Using the social media intervention design framework, which aims to adapt behavioral intervention for the social media delivery [30], this structured and systematic approach guided the development and implementation of the intervention. The primary objective of this intervention is to improve the student’s KAB about the SIV.

The WeChat intervention in this study was developed and designed following the cocreation approach [31]. Involving collaborative efforts between academic researchers and end users has great potential to enhance the customization of interventions for specific end users and settings [31-33]. The cocreation approach has been widely applied in other health interventions, especially for hard-to-reach participants [34,35]. Inspired by previous research, we initiated a cross-sectional survey grounded in the Capability, Opportunity, and Motivation Behavior model and Theoretical Domain Framework [36,37]. The survey results suggested that a lack of knowledge is the main barrier to improving SIV uptake [37].

Following the survey, we conducted 3 rounds of focus groups within a 2-month timeframe, involving 11 volunteer students. Each focus group had a specific objective. The first group was tasked with selecting the most suitable content and style for the intervention. Over the subsequent 2 weeks, the research team, in collaboration with 2 student helpers, curated a content pool based on the feedback received. The second focus group was convened to gather feedback and enhance the finer details of the selected content. This feedback-driven refinement process was crucial to ensuring the quality and relevance of our intervention materials. After these improvements, the final focus group was convened to evaluate the readability and usability of the intervention content and associated functions. This step was pivotal in ensuring that our intervention would be effective and user-friendly.

**Figure 1:**
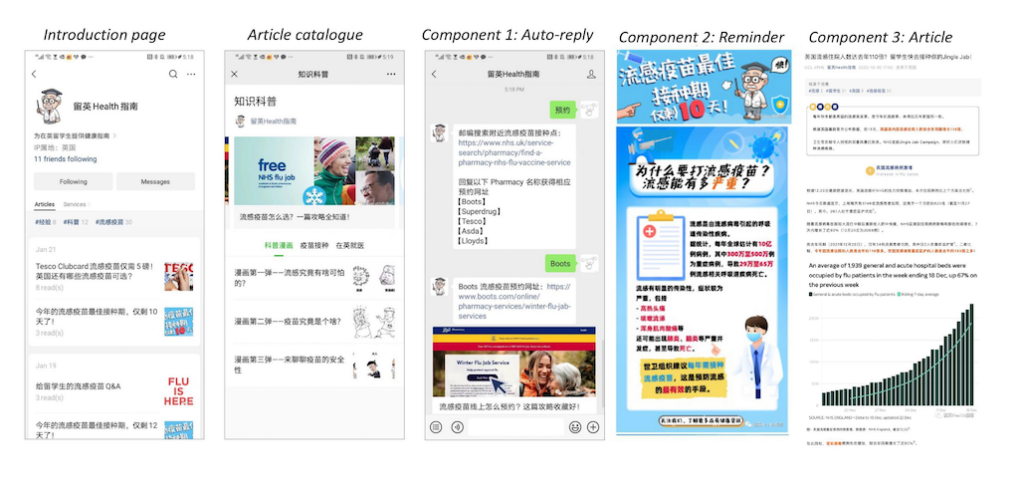
WeChat official account intervention (screenshots of the example pages).

The process for designing and developing this intervention is outlined in detail in our previous publication [38]. The final intervention comprised 3 main components: educational posts, an autoreply function, and reminders. Educational posts provided scientifically validated information about the importance of vaccination, accessible vaccination locations, and discounted vaccine options. The autoreply function addressed access barriers by offering personalized responses to inquiries about vaccination, using a predefined set of keywords. Reminders were strategically paired with educational content to prompt timely vaccination actions, supported by visual aids illustrating the optimal vaccination period. This multicomponent approach aimed to enhance informed decision-making and promote influenza vaccine uptake among the target population. Figure 1 presents visual representations of these components.

During the 4 weeks of the intervention phase, the intervention group received personal reminders 3 times a week (at 5 PM GMT on Monday, Wednesday, and Friday) and was exposed to the intervention contents and functions during the whole period, whereas the comparison group was blinded to any intervention materials and accesses.

### Participants and Recruitment

**Figure 2:**
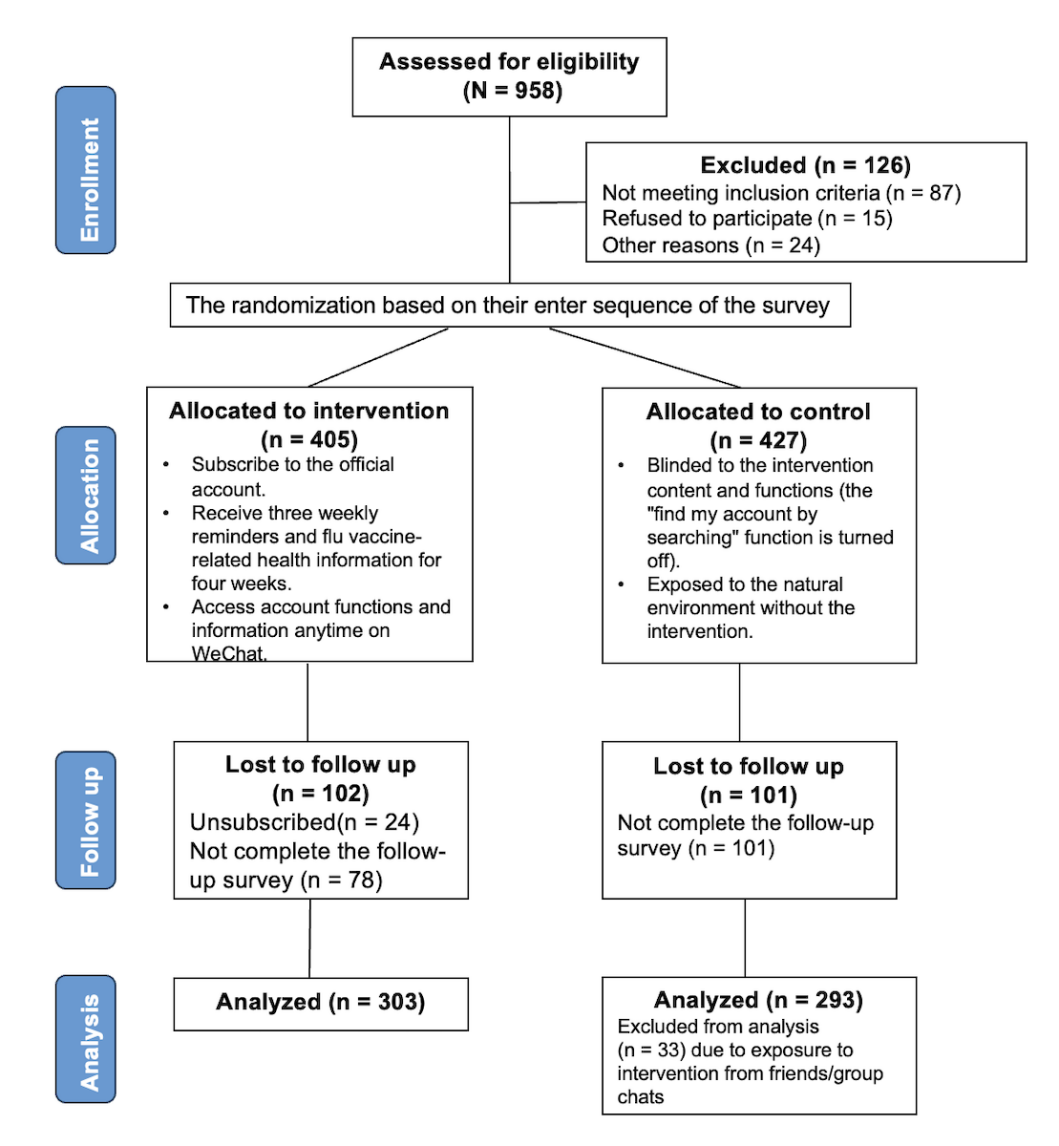
Overview of the design with baseline assessment, intervention, and follow-up assessments in the intervention and control groups.

The inclusion criteria for participants were as follows: (1) aged >18 years, (2) currently studying in a UK university, (3) international students from mainland China, (4) native Mandarin speaker, and (5) familiarity with using the WeChat official account to obtain information and communicate with others. No restrictions were set on universities and cities, as no significant differences were found in influenza vaccine settings and attitudes and behaviors based on the background survey [37]; therefore, all universities in the United Kingdom were included to maximize the sample size. The participants who met the criteria were invited to fill out the baseline web-based survey before the intervention. At the end of the baseline survey, the participants were randomly invited to subscribe to the WeChat official account. The chance of receiving the invitation has been predefined as 1:1. The participants who subscribed to the official account were then labeled as the intervention group. Figure 2 provides recruitment details.

To recruit the participants, advertisements were posted through a poster that emphasized the financial incentive and provided a point of contact for questions. The content of the posters only briefly mentioned the questionnaire content with the sentence in Chinese: “Have you received the influenza vaccination in the UK? Tell us what you think about it!” which was intentionally framed in a neutral manner, focusing on participants’ personal experiences rather than steering their perceptions in any particular direction. Once participants accessed the questionnaire, they were presented with an information consent sheet. This document detailed the broader purpose of the study, framed the intervention as an opportunity to receive evidence-based information about the influenza vaccine, and emphasized the voluntary base of the study. The financial incentives were guaranteed and were not influenced by whether participants used the intervention or received the vaccine. The language used in this stage was carefully crafted to avoid priming toward specific outcomes or opinions.

The study recruited participants through popular Chinese social media platforms, including WeChat, Weibo, and Red (Little Red Book). The research team disseminated the poster through their account and accessible chat groups and channels. Snowball sampling was adopted to increase the number of participants. Participants were given CNY ¥10 (US $1.40) as a reward for completing the initial survey. Double the amount was offered as an incentive for completing the survey after the intervention period. A bonus of CNY ¥5 (US $0.70) was given for referring another eligible participant to complete the initial survey.

### Data Collection and Outcomes

#### Overview

Baseline and follow-up data were gathered through questionnaires administered in early December 2022 and late January 2023, representing the periods before and after the implementation of the intervention. The assessment of sociodemographic characteristics and KAB scores was conducted using a web-based survey at both the baseline and postintervention stages. In the follow-up survey, user feedback was specifically solicited from the intervention group, while questions regarding exposure to the intervention content were posed to the control group. This approach minimized the risk of data contamination. To maintain data integrity, participants in the control group who were inadvertently exposed to the intervention were excluded from the analysis. In addition, students in both groups who reported having received the SIV in the baseline survey were excluded from the study. Detailed questionnaires can be found in Multimedia Appendix 1*.*

#### Primary Outcome: KAB Score

The influenza vaccination KAB of university students were assessed at baseline and at a 4-week follow-up using a 26-item questionnaire, as presented in Textbox 1. The questionnaire included three sections: (1) knowledge section (10 questions picked randomly from the question pool and 6 self-rated scores; for each knowledge point, a question pool was created with 5 identical questions, and 1 question was randomly selected to ask participants), which was related to the importance of preventing influenza, the benefits of taking SIV, and how to get vaccinated in the United Kingdom; (2) vaccine attitude section (14 questions), which included statements about SIV with 5-point Likert-scale options; and (3) vaccination behaviors’ section (2 questions): in this section, we first asked whether the participant is vaccinated, if the answer is “no,” they will be directed to answer the intended behavior question with 5 options. The KAB-related questions were either formulated or adapted from published questionnaires and documents from local health promotion centers in the United Kingdom and China [39-41]. All questions were reviewed by 2 experts with experience in vaccination-related survey research and were also pretested with 30 students to ensure accuracy, clarity, and cultural adequacy. Minor modifications were made to the questions according to feedback from students.

The questions on vaccination KAB were separately analyzed. For the knowledge questions, each correct response was allocated a score of 1 point, and an incorrect or no response was allocated 0 point. The total knowledge score ranged between 0 and 10 points, with higher scores indicating that the student displayed better SIV knowledge. The self-rated knowledge scores were analyzed to assess the knowledge score reliability. For the attitude statements, each question was measured on a 5-point Likert scale (strongly disagree=1, disagree=2, moderate=3, agree=4, and strongly agree=5). The average score of the 14 attitudes was calculated as the attitude score (range 1-5), whereby the favorable options (strongly agree) were given 5 points each and the unfavorable options (strongly disagree) were given 1 point. Reverse coding has been applied to the items rephrased negatively. A higher attitude score reflected a more positive attitude toward SIV. For the question related to vaccination behavior, the actual behavior was coded as “unvaccinated=0” and “vaccinated=1,” and the intended behavior scores were from 1 to 5 (not at all=1, unlikely=2, not sure=3, likely=4, and very likely=5). Details related to each of the KAB questions and the coding of correct answers are presented in Multimedia Appendix 2.

**Knowledge, attitude, and behavior instruments.**

**Knowledge**

1. At-risk group (eg, older adults, infants and young children, and people with chronic illnesses are at high risk of influenza [single choice] [I think this statement is (correct, wrong, or not sure)]
2. Influenza susceptibility (eg, all people are generally susceptible to influenza [single choice] [I think this statement is (correct, wrong, or not sure)]
3. Flu symptoms: (eg, the typical symptoms of influenza include cough, sore throat, runny nose, and nasal congestion [single choice] [I think this statement is (correct, wrong, or not sure)]
4. Timing of vaccination (eg, in the United Kingdom, the best time to get an influenza vaccination is between October and January [single choice] [I think this statement is (correct, wrong, or not sure)]
5. Preventive measures for influenza (eg, the preventive measures for influenza are similar to those for the COVID-19 [single choice] [I think this statement is (correct, wrong, or not sure)]
6. Flu severity (eg, the number of hospitalizations due to influenza is about 3 to 5 million worldwide each year [single choice] [I think this statement is (correct, wrong, or not sure)]
7. Where to get the vaccine (eg, in the United Kingdom, the only way to get an influenza vaccination is to go to the hospital [single choice] [I think this statement is (correct, wrong, or not sure)]
8. Vaccination frequency (eg, you need to get a one-shot influenza vaccination annually [single choice] [I think this statement is (correct, wrong, or not sure)]
9. Who need flu vaccination (eg, only people at high risk need to be vaccinated against influenza [single choice] [I think this statement is (correct, wrong, or not sure)]
10. Flu vaccine benefits (eg, you will not catch the influenza if you get the influenza vaccine [single choice] [I think this statement is (correct, wrong, or not sure)]

**Attitude**

1. I believe the influenza vaccine is safe.
2. I believe the influenza vaccine is effective.
3. The influenza vaccine is important for my health.
4. I believe that the process of producing and administering influenza vaccines is safe and effective.
5. The information promoted by the government, universities, and the National Health Service about the influenza vaccine is trustworthy.
6. If I do not get the influenza shot, I may catch the influenza.
7. I think that the natural immunity that comes from having the influenza is better than the influenza shot.
8. I think that the probability of me getting the influenza is so low that I do not need the vaccine.
9. I think even if I get the influenza I can live with it, so I do not need the vaccination.
10. The place and environment for receiving vaccinations in the United Kingdom is so bad that it makes me not want to get vaccinated.
11. The processes of making appointments and receiving the influenza vaccinations in the United Kingdom are easy to me and take little time.
12. I believe I will be able to get the influenza vaccine if needed.
13. I can afford the influenza vaccine cost.
14. I always be proactive in keeping up with information about the influenza vaccine.

**Behavior**

1. Have you received the influenza vaccination since October 1, 2022? [single question, yes or no; refers to actual behavior]
2. How likely are you to receive the influenza vaccine within the next 3 months? [single question; not at all, unlikely, not sure, likely, or very likely; refers to intended behavior]

#### Secondary Outcomes: User Data, Reviews, and Scores

User data, including the number of reads, users, and click-throughs, were automatically collected through WeChat each time a user accessed the official account. Time stamps were recorded for these interactions. The collected user engagement data were then extracted and exported to the CSV format. These data serve as the secondary outcomes for assessing the popularity and usability of the intervention.

To gauge users’ satisfaction with the intervention, a set of 10 statement questions was adapted from the System Usability Scale [42]. Participants in the intervention group were asked to respond to these questions using a 5-point Likert scale at the end of the follow-up survey, providing insights into their feelings about the content and overall effectiveness of the intervention. At the end of the survey, participants were invited to input their feedback and suggestions regarding the intervention. This qualitative information helps in understanding user perspectives.

#### Sample Size Calculation

As the attitude and behavior scores are binary or ordinal measurements, the knowledge score, being the only numeric variable, was used for calculating the required sample size for the intervention study. On the basis of the pilot study results and previous research, we assumed at least a 1-point improvement in the knowledge score postintervention [43,44]. A larger SD of 5 was used to account for potential discrepancies among participants.

The sample size calculation was performed using the hypothesis test method to ensure narrow CIs with high reliability. This calculation was based on a priori analysis with 95% power and an α of .05. We anticipated a dropout rate of no more than 30% for both groups. To accommodate this, a baseline sample size of 950 participants was estimated to provide sufficient power for testing our hypotheses, considering the expected dropout rate. A paired 2-tailed *t* test was selected under the assumption of normality, and the following function was used to calculate the minimum number of pairs of participants needed [45,46]:




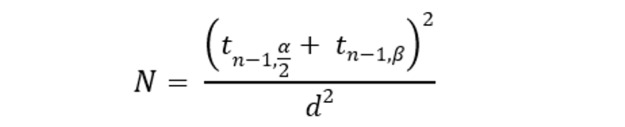

**(1)**



where 
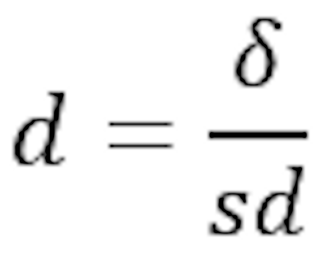
, α=alpha, β=1–power, and *t* is a *t* test quantile with *n–1* df and 
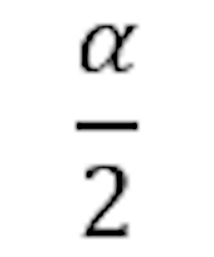
 probability. *N* was rounded up to the closest integer.

### Data Analysis

Descriptive statistics were first used, presenting continuous variables as means with SDs and categorical variables as frequencies with proportions. To assess the baseline characteristics of participants between the intervention and control groups, clustered independent *t* tests and χ^2^ analyses were used for sociodemographic and vaccine-related measures.

The normality of the KAB scores was initially assessed using Shapiro-Wilk tests. Given that the data exhibited skewness, paired Wilcoxon signed rank tests were subsequently used. These tests independently compared the differences in KAB scores between baseline and the 4-week follow-up within each of the intervention and control groups, focusing on within-group differences. In addition, clustered independent Wilcoxon signed rank tests were used to evaluate between-group differences (intervention vs control groups) in mean score changes (ie, follow-up minus baseline scores). To explore the interplay between KAB scores, a series of correlation tests were first conducted. Subsequently, path analysis was conducted to explore potential mediation and causal effects among the knowledge, attitude, intention, and behavior scores and group difference; the results were evaluated using the following indices with the threshold for a perfect fit: chi-square goodness of fit (χ^2^), root mean square error of approximation (RMSEA; <0.10), goodness of fit index (GFI; ≥0.90), root mean square residuals (RMR; ≤0.05), normed fit index (NFI; ≥0.90), non-normed fit index (NNFI; ≥0.90), and comparative fit index (CFI; ≥0.90) [47]. Multiple linear regression analyses were performed to assess the mean changes in KAB scores while adjusting for covariates that exhibited significant differences at baseline between the intervention and control groups.

To assess satisfaction with the WeChat official account intervention among participants in the intervention group, descriptive statistics were used. Qualitative data were analyzed through keyword frequency analysis [48]. Use data collected by the WeChat system were extracted, and temporal analysis was carried out using line graphs. All data analysis and visualization were conducted using R Studio Version 2023.06.1+524, with a significance level of *P*<.05 considered statistically significant.

### Ethical Considerations

The authors affirm that all procedures in this study adhere to ethical standards outlined in the Helsinki Declaration of 1975, as revised in 2008, and were approved by the University College London Ethics Committee (21647/001). All human participants provided informed consent before participating in the survey, and this consent was digitally recorded on the survey platform. It is important to note that this study did not involve minors.

## Results

### Sociodemographic Details

The study sample comprised 596 students, with a mean age of 22.5 (SD 3.345) years and an age range spanning from 18 to 38 years. The gender distribution approached parity, with 51.7% (308/596) identifying as male and 48.3% (288/596) as female. Most participants, exceeding 81.5% (486/596), pursued their studies in London, and 94.8% (565/596) were enrolled in academic programs unrelated to the medical field. In terms of group differences, a statistically significant age difference was observed between the intervention and control groups, with students in the intervention group being slightly older. In addition, a noticeable disparity in educational backgrounds emerged, with a higher proportion of postgraduate students in the intervention group (181/303, 59.7%) compared to their undergraduate counterparts in the control group (189/293, 64.5%). Regarding their past vaccination history, the intervention group showed a lower rate of past influenza vaccination (60/303, 19.8% vs 82/293, 27.9%; *P*=.02) but a higher rate of full COVID-19 vaccination (264/303, 87.1% vs 266/293, 90.8%; *P*=.002). In this baseline survey, participants from the intervention group reported a significantly lower average attitude score toward SIV, while no significant difference was found in the knowledge and behavior scores between the groups. Table 1 displays the sociodemographic details for the 2 groups.

**Table 1:** Baseline sociodemographic and vaccination characteristics of the university students enrolled in the intervention and control groups (N=596).

Characteristics				Total sample		Intervention (n=303)		Control (n=293)		*P* value^a^	
**Sociodemographic characteristics**											
	Age (y), mean (SD)			22.532 (3.345)		23.531 (3.708)		21.498 (2.550)		<.001	
	**Sex, n (%)**										.22
		Male	308 (51.7)		164 (54.1)		144 (49.1)				
		Female	288 (48.3)		139 (45.9)		149 (50.9)				
	**Education, n (%)**										<.001
		Undergraduate	311 (52.2)		122 (40.3)		189 (64.5)				
		Postgraduate	285 (47.8)		181 (59.7)		104 (35.5)				
	**City, n (%)**										.006
		London	486 (81.5)		260 (85.8)		226 (77.1)				
		Others	110 (18.5)		43 (14.2)		67 (22.9)				
	**Major, n (%)**										.93
		Medical related	31 (5.2)		16 (5.3)		15 (5.1)				
		Non–medical related	565 (94.8)		287 (94.7)		278 (94.9)				
**Health,** **n (%)**											
	**Self-reported health status**										.001
		Very good	340 (57)		147 (48.5)		193 (65.9)				
		Good	215 (36.1)		129 (42.6)		86 (29.4)				
		Fair	37 (6.2)		25 (8.3)		12 (4.1)				
		Poor	2 (0.3)		1 (0.3)		1 (0.3)				
		Very poor	2 (0.3)		1 (0.3)		1 (0.3)				
	**Influenza infection history**										.61
		Yes	407 (68.3)		204 (67.3)		203 (69.3)				
		No	189 (31.7)		99 (32.7)		90 (30.7)				
**Vaccination, n (%)**											
	**Past influenza vaccination**										.02
		Yes	142 (23.8)		60 (19.8)		82 (27)				
		No	454 (76.2)		243 (80.2)		211 (72)				
	**Annually influenza vaccination habit**										.07
		Yes	35 (5.9)		23 (7.6)		12 (4.1)				
		No	561 (94.1)		280 (92.4)		281 (95.9)				
	**COVID-19 vaccine history**										.002
		Completely vaccinated	530 (88.9)		264 (87.1)		266 (90.8)				
		Partially	53 (8.9)		26 (8.6)		27 (9.2)				
		None	13 (2.2)		13 (4.3)		0 (0)				
**KAB^b^ score^c^, mean (SE)**											
	Knowledge			5.822 (0.073)		5.756 (0.098)		5.891 (0.109)		.36	
	Attitude			3.625 (0.019)		3.564 (0.026)		3.688 (0.028)		.001	
	Behavior (intended)^d^			2.463 (0.039)		2.475 (0.049)		2.451 (0.061)		.75	

^a^A comparison of baseline characteristics between intervention and control groups was conducted for continuous and categorical variables using clustered independent *t* and χ^2^ tests. Statistical significance was determined at *P*<.05.

^b^KAB: knowledge, attitude, and behavior.

^c^The total knowledge and attitude scores ranged from 0 to 10 points and 1 to 5 points, respectively, and the behavior (intended) score ranged from 1 to 5 points.

^d^The intended behavior score is measured by vaccination intention: not at all=1, unlikely=2, not sure=3, likely=4, and very likely=5.

### Primary Outcome

**Figure 3:**
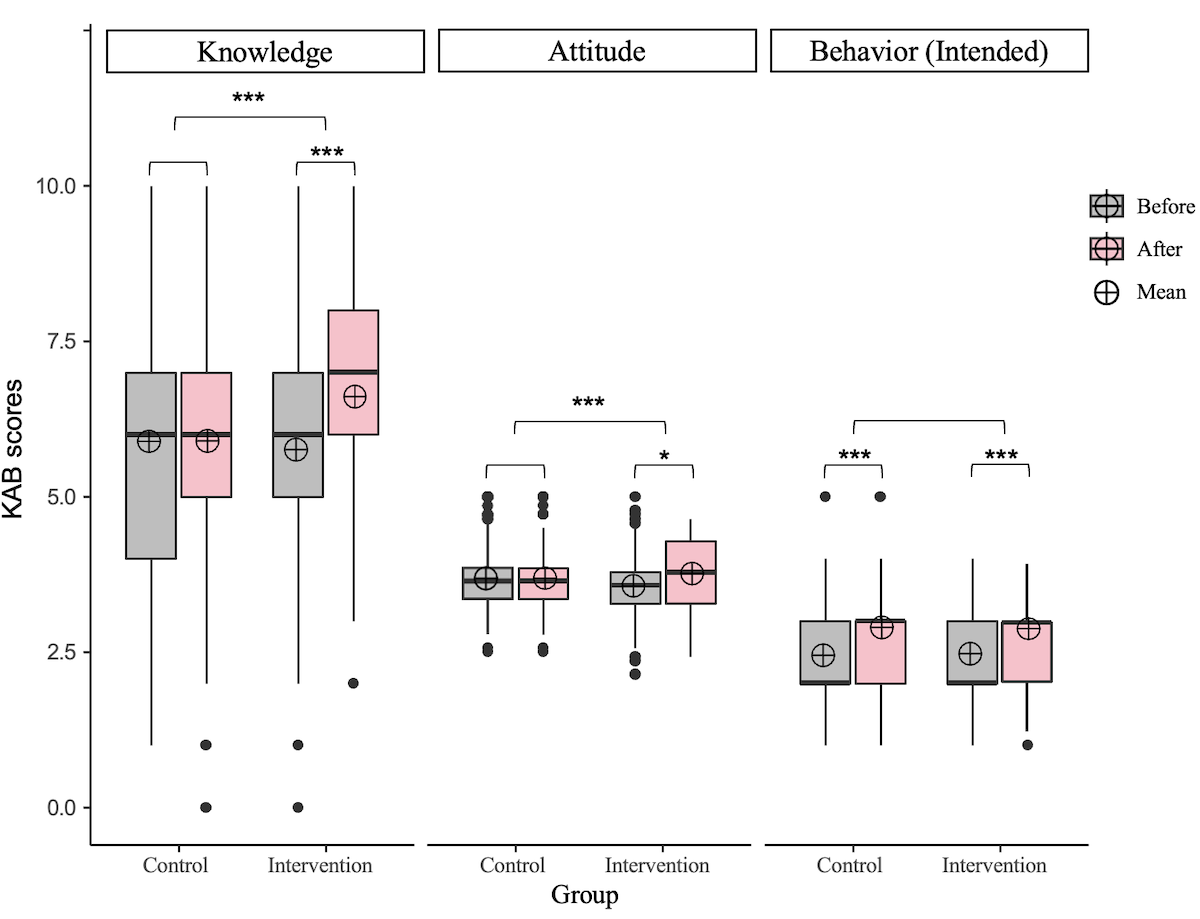
Comparing between- and within-group differences in knowledge, attitude, and behavior scores with statistical significance (statistical significance was determined using clustered Wilcoxon signed rank tests, while between-group differences were assessed by comparing within-group variations). Only significant results (*P*<.05) were marked: *P=.01-.05, ***P<.001.

When examining changes over time within each group, significant improvements were evident in KAB scores within the intervention group (*P*<.001). In contrast, the control group displayed only a slight increase in intended behavior scores (*P*<.001). When evaluating changes over time between the 2 groups by comparing the differences in within-group changes, the intervention group exhibited significant differences in knowledge scores compared to the control group (mean 0.845, SE 0.071 vs mean 0.010, SE 0.048, respectively; *P*<.002). Specifically, at baseline, the mean knowledge score for the intervention group was 5.756 (SE 0.098), while the control group had a mean score of 5.891 (SE 0.109). There was no significant difference between the groups at baseline (*P*=.36). At follow-up, the intervention group’s mean knowledge score increased to 6.604 (SE 0.102), whereas the control group’s mean score increased to 5.901 (SE 0.116). These results indicate that the intervention was effective in significantly improving participants’ knowledge about the influenza vaccine compared to the control group. Similarly, the increase in attitude scores for the intervention group was significantly higher compared to the control group (mean 0.199, SE 0.041 vs mean 0.002, SE 0.004, respectively; *P*<.001). However, there was no significant difference in intended behavior scores between the intervention and control groups (mean 0.894, SE 0.058 vs mean 0.877, SE 0.066; *P*=.62). In terms of actual behavior, the vaccination rate in the intervention group (63/303, 20.8%) was slightly higher than that in the control group (54/293, 18.4%). Figure 3 illustrates the within- and between-group differences, presenting the mean change from baseline to the 4-week follow-up in KAB scores for the study sample. Detailed statistical information is provided in Multimedia Appendix 3.

We conducted a series of correlation tests to examine the interplay between postintervention knowledge, attitude, intended and actual behavior, as well as group differences. These tests revealed significant correlations: knowledge score and intended behavior (correlation coefficient=0.63, 95% CI 0.57-0.68; *P*<.001), attitude score and intended behavior (correlation coefficient=0.13, 95% CI 0.04-0.22; *P*=.004), knowledge score and actual behavior (correlation coefficient=0.36, 95% CI 0.29-0.43; *P*<.001), attitude score and actual behavior (correlation coefficient=0.21, 95% CI 0.14-0.29; *P*<.001), and intended behavior and actual behavior (correlation coefficient=0.77, 95% CI 0.74-0.90; *P*<.001).

**Figure 4:**
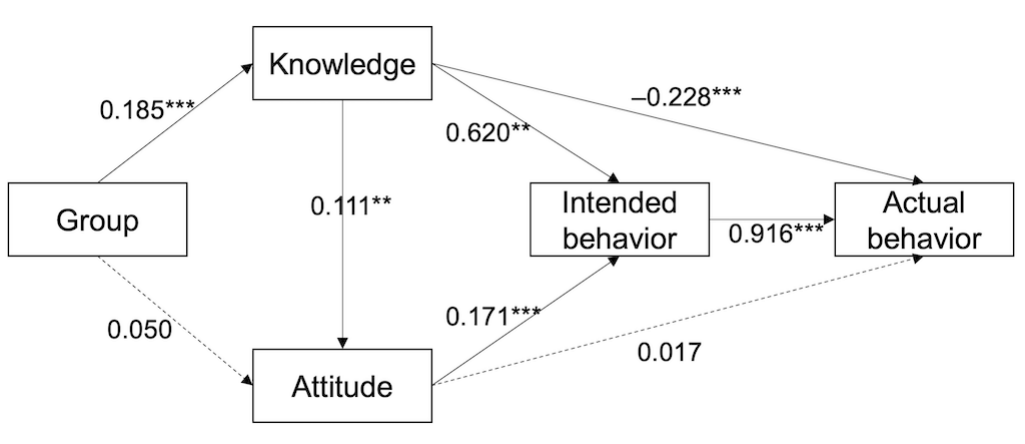
Path diagram, indicating the relationships between variables (numbers along the arrows indicate path coefficients, which reflect the strength and direction of the relationship between variables. **P=.01-.001, ***P<.001.

As correlation analysis cannot establish causal relationships among variables, we used path analysis. We initially developed a theoretical model based on prior literature, positing that the intervention affects intended behavior by altering both knowledge and attitude. Intended behavior, in turn, has a direct impact on actual behavior [49,50]. Knowledge and attitude may also directly influence actual behavior. Consequently, we constructed a structural equation model encompassing these relationships. Model fit indices, including χ^2^, GFI, RMSEA, RMR, CFI, NFI, and NNFI, were considered for model adjustment and improvement. The final adjusted model demonstrated satisfactory fit (GFI=0.986, RMSEA=0.074, RMR=0.015, CFI=0.987, NFI=0.983, and NNFI=0.963). The causal relationships and path coefficients are illustrated in Figure 4.

In the path analysis, the group has a significant, direct impact on knowledge, with a path coefficient of 0.185 (*P*<.001), confirming the intervention’s direct influence on knowledge. However, it does not directly affect attitudes, as indicated by an insignificant path coefficient of 0.050 (*P*=.23). In addition, a notable and positive cause-and-effect relationship is observed between knowledge and attitude, which implies that knowledge plays an immediate and substantial role in shaping students’ attitudes toward vaccination. Both knowledge and attitude are found to have direct and significant associations with intended behavior, as denoted by path coefficients of 0.620 and 0.171 (both *P*<.01). This demonstrates that higher levels of knowledge and more favorable attitudes are associated with a stronger intention to receive the vaccine. Furthermore, it is worth noting that there exists a significant and positive correlation between intended behavior and actual behavior, as indicated by a path coefficient of 0.916 (*P*<.001), which underscores the strong connection between students’ intentions and their subsequent actions to vaccination. Interestingly, an inverse relationship is identified between knowledge and actual behavior. Specifically, students with higher knowledge scores tend to exhibit less engagement in actual vaccine-receiving behavior.

Table 2 presents the results derived from the general linear regression models, which investigate the factors influencing the differences in KAB scores within both the intervention and control groups. Notably, within the intervention group, significant associations were found in relation to gender and self-reported health status in the domain of knowledge scores. Male participants exhibited a noteworthy decrement in knowledge scores (–0.326; *P*=.02) in comparison to female participants, while self-reported health status displayed a negative correlation (–0.235; *P*=.02) with knowledge score change, indicating that higher self-reported health scores corresponded to lower knowledge improvement. In contrast, the control group did not exhibit statistically significant associations with any of the factors in the domain of knowledge or attitude scores. Conversely, concerning changes in intended behavior scores, several significant associations emerged within the intervention group. Gender (*P*=.001), self-reported health status (*P*=.04), past influenza vaccination (*P*<.001), annual influenza vaccination habits (*P*=.02), and COVID-19 vaccine acceptance all demonstrated statistically significant relationships with mean intended behavior score changes. Specifically, female participants, those with lower self-reported health statuses, those who have received past influenza shots, and those with annual influenza vaccination practices exhibited higher change in intended behavior scores, indicative of increased intentions of receiving SIV. In the control group, only the influenza infectious displayed significant associations with intended behavior scores. These findings highlighted the differential impact of sociodemographic and health-related factors on KAB scores within the intervention and control settings.

**Table 2:** General linear regression models for mean change in knowledge, attitude, and behavior scores.

Categories^a^	Change in knowledge scores, mean (95% CI)		Change in attitude scores, mean (95% CI)		Change in behavior scores (intended), mean (95%CI)	
	Intervention	Control	Intervention	Control	Intervention	Control
Age	0.014 (–0.023 to 0.052)	0.008 (–0.094 to 0.109)	−0.013 (–0.034 to 0.009)	0.004 (–0.004 to 0.0012)	0.051 (0.016 to 0.085)	–0.011 (–0.046 to 0.024)
Male sex	–0.326 (–0.604 to –0.049)^b^	0.062 (–0.126 to 0.25)	−0.02 (–0.182 to 0.142)	–0.011 (–0.026 to 0.005)	–0.492 (–0.719 to –0.265)^c^	–0.221 (–0.530 to 0.088)
Undergraduate education	0.081 (–0.203 to 0.366)	–0.014 (–0.211 to 0.183)	0.054 (–0.11 to 0.219)	–0.011 (–0.027 to 0.005)	–0.319 (–0.554 to 0.083)	–0.011 (–0.339 to 0.317)
Self-reported health status	–0.235 (–0.439 to –0.031)^b^	–0.132 (–0.283 to 0.019)	–0.109 (–0.227 to 0.01)	0.003 (–0.01 to 0.015)	–0.184 (–0.359 to –0.009)^b^	–0.211 (–0.449 to 0.027)
Influenza infectious	–0.08 (–0.378 to 0.217)	0.047 (–0.158 to 0.252)	0.002 (–0.171 to 0.174)	0.001 (–0.016 to 0.018)	0.049 (–0.203 to 0.301)	0.034 (–0.293 to 0.361)^c^
Past influenza shot	0.326 (0.023 to 0.62)	0.099 (–0.11 to 0.308)	0.16 (–0.042 to 0.361)	–0.001 (–0.018 to 0.016)	1.175 (0.905 to 1.445)^d^	0.703 (0.362 to 1.044)
Annual influenza vaccine habit	0.209 (–0.318 to 0.736)	0.163 (–0.311 to 0.638)	0.14 (–0.164 to 0.445)	0.004 (–0.035 to 0.043)	0.575 (0.091 to 1.058)^b^	0.601 (0.380 to 1.582)
COVID-19 vaccine	–0.105 (–0.604 to 0.394)	0.052 (–0.273 to 0.377)	–0.015 (–0.304 to 0.274)	0 (–0.02 to 0.026)	1.008 (0.353 to 1.663)^c^	0.438 (–0.062 to 0.938)

^a^We excluded major and city from our analysis due to imbalanced sample sizes.

^b^.01*<P*<.05.

^c^.001*<P*<.01.

^d^*P*<.001.

### Secondary Outcome

**Figure 5:**
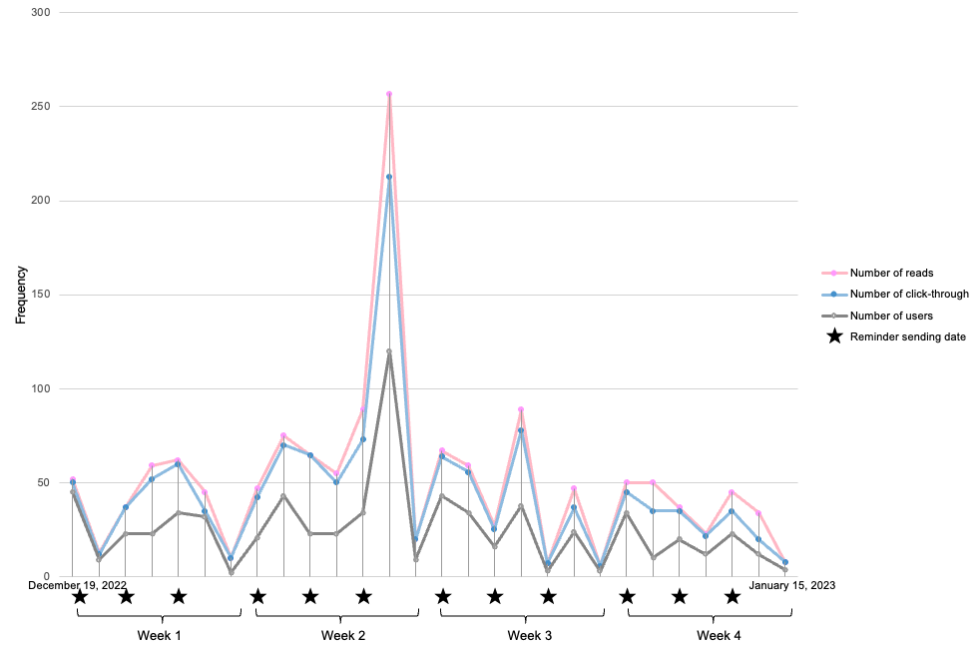
Temporal trend of reads, users, and click-throughs during the 4-week intervention period.

Throughout the 4-week intervention period, the daily averages for both reads and click-throughs were 51 and 26, respectively. This temporal trend is illustrated in Figure 5, where the numbers of reads, users, and click-throughs exhibit a consistent pattern. Notably, these metrics displayed an upsurge in reminder posting dates and the subsequent days. The peak day, observed on Saturday during week 2, coincided with the translation and reposting of a news article from National Health Service England addressing the escalating hospital crises attributed to a surge in influenza cases and promoting the “Jingle Jab” campaign, which encouraged students to get vaccinated during the Christmas holiday.

In terms of use frequency, a significant portion of the participants engaged with the platform weekly (120/303, 39.6%), and a notable minority used it daily (33/303, 10.9%), while others turned to it on an as-needed basis. The primary function used by most users was reading articles (234/303, 77.2%), followed by using the autoreply feature to seek answers to their queries (109/303, 35.9%). In addition, 26.4% (80/303) of participants reported that they had shared or mentioned articles or accounts with others.

**Figure 6:**
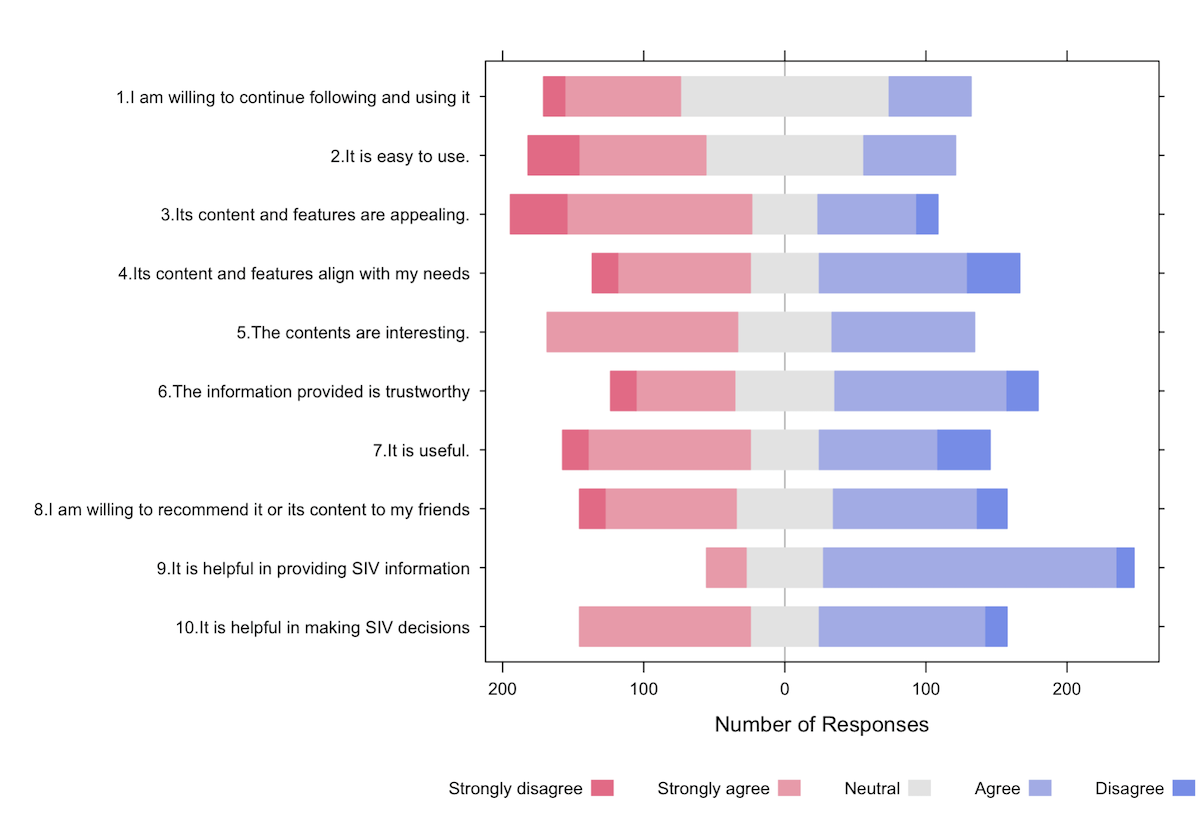
Likert chart displaying the participant responses on user satisfaction with the WeChat official account. SIV: seasonal influenza vaccination.

In general, participants expressed a high level of satisfaction with the WeChat official account. Specifically, 78.9% (240/303) of participants expressed their willingness to continue following and using this official account, while 66.3% (201/303) stated that they would recommend it to their friends. In terms of the content disseminated by the account, >60% of participants either agreed or strongly agreed that the content was trustworthy (236/303, 77.9%), appealing (195/303, 64.4%), interesting (192/303, 63.4%), and relevant to their needs (199/303, 65.7%). In addition, more than half of the participants affirmed that the intervention through WeChat official accounts effectively provided information about SIV and supported their decision-making process. Figure 6 provides a detailed breakdown of participant responses regarding user satisfaction. Detailed statistical information is provided in Multimedia Appendix 4.

In the qualitative feedback, the term “useful” was the most frequently mentioned. In addition, “professional” and “rigorous” were also commonly cited attributes. In the open-ended question, participants noted that the inclusion of detailed source references with each article enhanced the credibility of the information provided. Among the most memorable articles, those related to the influenza epidemic in the United Kingdom garnered the highest readership, followed by articles discussing influenza vaccine discounts and instructions for booking appointments. This finding aligns closely with the data recorded by the system, confirming the high engagement with these topics.

Participants also provided several suggestions for improvement. For example, participants suggested incorporating more emotional content in articles to make them more engaging. There were requests for comparative information between Chinese and British influenza vaccines. Participants expressed a desire for information outlining measures to take after contracting influenza. Suggestions were made to increase promotional efforts to enhance the visibility and reach of the information. In addition, participants highlighted the limitations of disseminating relevant information on WeChat. They noted challenges such as users’ numerous subscriptions to various public accounts, which made it less likely for them to notice updates from this particular account. Furthermore, medical information was perceived as having limited appeal, with users primarily accessing it only when a specific need arose.

## Discussion

### Principal Findings

This study aimed to evaluate the effectiveness of a WeChat-based intervention in improving KAB regarding SIV among mainland Chinese university students in the United Kingdom. The intervention was designed using a cocreation approach and was delivered through WeChat, incorporating educational posts, an autoreply function, and reminders. The primary findings of the study indicate that the WeChat-based intervention significantly improved participants’ knowledge and attitude about SIV. The mean knowledge and attitude scores increased significantly in the intervention group compared to the control group, highlighting the efficacy of the intervention in enhancing students’ understanding of SIV and influencing their attitudes toward it.

### Key Findings and Comparison With Prior Work

Numerous studies have underscored the crucial role of social media in disseminating vaccine-related knowledge and facilitating shifts in attitudes [13,14,16]. This stems from the inherent nature and function of social media platforms, which enable the sharing and reception of information, while also offering a means of social support in the context of vaccines [14,51]. The positive results could also be explained by the adoption of a theory-driven and cocreation approach in designing the intervention, which increased the engagement and usability of the information delivered. Prior research found that catchy visuals are required for promoting influenza vaccine among university students, highlighting the importance of the co-design method in designing the intervention content [52]. However, it is noteworthy that the changes in behavior scores are identical between the intervention and control groups, suggesting that the intervention lacks sufficient evidence to induce behavioral change. This observation may be partly attributed to external factors, notably the occurrence of a severe influenza season in the United Kingdom that coincided with our intervention [2]. Consequently, the National Health Service initiated extensive campaigns to promote influenza vaccinations, aiming to alleviate the health care system’s strain [53]. The influence of this potential confounding factor cannot be discounted in our evaluation.

The path analysis results presented in this study reveal a comprehensive understanding of the intricate relationships among variables related to vaccine knowledge, attitude, intended behavior, and actual behavior. Our analysis highlights a significant and direct effect of the intervention on knowledge. As a crucial initial step, it emphasizes the intervention’s role in enhancing knowledge regarding vaccines. Its influence on intended behavior is mediated through changes in both knowledge and attitude, which supports the assumption in our theoretical model. Knowledge and attitude together play a significant role in shaping students’ intentions to receive the vaccine. Previous studies also found that a higher level of knowledge and a more positive attitude are associated with a greater intention to receive the vaccine [54]. In addition, the substantial correlation between intended and actual behavior highlights the predictive power of students’ intentions in determining their subsequent actions, which differs from previous research [12]. Furthermore, the unexpected negative association between knowledge and actual behavior, where higher knowledge scores correspond to less actual behavior, is a compelling finding. While improved knowledge might lead to a reduced perception of the risk and severity of influenza, potentially explaining lower vaccine uptake [6], further investigation is needed to identify the underlying causes.

In terms of the sociodemographic factors, gender and self-reported health status emerged as significant determinants of knowledge score changes within the intervention group. These findings underscore the need for tailored interventions that consider demographic and health-related factors to effectively target knowledge enhancement. Intriguingly, several factors were significantly associated with changes in intended behavior scores within the intervention group, including gender, self-reported health status, past influenza vaccination, and annual influenza vaccination habits. Older students, female participants, those with lower self-reported health statuses, students with a history of past influenza vaccination, and those with established annual influenza vaccination practices exhibited higher change of intended behavior scores, indicating a greater increase of intention to receive the SIV. This suggests that future interventions should be customized to address these sociodemographic and health-related factors to maximize their impact.

In addition to evaluating KAB scores, our analysis extended to the examination of user engagement and satisfaction with the intervention platform. Over the 4-week intervention period, users consistently engaged with the platform, with activity peaks coinciding with our reminder postings. Users reported finding the content trustworthy, appealing, interesting, and highly relevant to their needs, indicating that the platform effectively delivered valuable information. Notably, the “content is interesting” metric received the highest score, underscoring the success of our collaborative content creation efforts in designing engaging materials that resonated with our users [31]. Moreover, the inclusion of specific source references enhanced the platform’s credibility, underscoring the significance of building public trust and credibility in vaccine promotion [55]. Notably, certain articles about the influenza epidemic in the United Kingdom attracted substantial readership, underscoring the role of factual information in heightening students’ awareness and interest in vaccination.

Students provided valuable suggestions for improvement. These included the integration of more emotionally resonant content, the provision of comparative information on vaccines, guidance on postinfluenza infection measures, and increased promotional efforts. Future interventions could incorporate such improvements. Furthermore, “easy to use” received the lowest positive feedback, with qualitative comments suggesting that users found the platform’s functionality somewhat overwhelming and less straightforward. For instance, users had to input specific keywords to access links, introducing a barrier to information retrieval. This feedback underscores the importance of streamlining the user experience and simplifying the platform’s functionalities to enhance user-friendliness and overall satisfaction. In addition, users noted challenges related to WeChat’s tendency to overwhelm users with information and the limited appeal of medical content. As a commercial platform, WeChat imposes certain limitations by restricting control over the platform’s features and functionality [23]. Considering the valuable insights gathered, future studies could explore the design of a new application that aligns more precisely with the specific intervention goals, if timing and budget constraints allowed. This proactive approach may address some of the functionality and engagement challenges identified in this evaluation.

### Strengths and Limitations

This study exhibits several notable strengths that enhance its methodological robustness. First, the study’s recruitment strategy harnessed the ubiquity and popularity of Chinese social media platforms, such as WeChat, Weibo, and Red. This approach facilitated the assembly of a diverse and expansive participant pool, providing a more comprehensive representation of students from mainland China. Second, the random assignment of participants to the intervention group was a pivotal methodological choice. This randomization process effectively mitigated the influence of selection bias. Moreover, the mixed methods design of the study, which incorporated the collection of user engagement data and user satisfaction feedback, added a layer of depth to the research. These additional data sources offered valuable insights into the intervention’s usability, thereby providing a more holistic understanding of its effectiveness.

However, it is imperative to acknowledge the limitations inherent in the study. First, the reliance on social media platforms for participant recruitment introduced the potential for sampling bias. This bias may stem from the tendency of more active platform users to participate, potentially limiting the generalizability of the findings to other student populations who may not be as active on these platforms. Future research may consider diversifying recruitment strategies to mitigate this potential limitation. Second, the use of snowball sampling is an important limitation of study quality. Snowball sampling, while useful for reaching hard-to-access populations, can lead to sampling bias and affect the representativeness of the sample. Participants tend to refer to individuals who are similar to themselves, which can result in a homogenous sample and limit the generalizability of the findings. Future studies should aim to use more systematic sampling methods to enhance the representativeness of the sample. Third, despite using random invitations for the intervention group, the control group may still comprise students who self-selected not to participate. This introduces the possibility of self-selection bias, as those who chose not to engage with the intervention may possess characteristics or preferences that differ systematically from those who did. This should be considered when interpreting the results. Fourth, the exclusive focus on international students from mainland China studying in the United Kingdom, while valuable for our research questions, could restrict the generalizability of the findings to a broader student population. The cultural and contextual factors unique to this subgroup may not completely align with the experiences of other student demographics, warranting caution when extrapolating the results to different populations. In addition, the study’s reliance on self-reported data presents potential challenges related to recall bias. Participants may be inclined to provide socially desirable responses or may exhibit inaccuracies in recall; thus, careful interpretation is necessitated.

Moreover, limitations associated with the WeChat platform itself may introduce bias into the results. Participants have reported difficulty in noticing reminders due to the multitude of official accounts they have subscribed to. Strategies such as sending reminders during off-peak hours in mainland China to maximize the likelihood of visibility may help mitigate this bias. Designing an application from scratch would be beneficial for future work as it could support a more personalized experience for research purposes. In addition, the timing of the study may have influenced the quality of responses. Conducted in the United Kingdom from December 2022 to January 2023, the study took place during the country’s most severe influenza season in 5 years. At the same time, China was experiencing a challenging COVID-19 situation and related policy changes, which heightened interest in health-related information on social media. Although the control group has been added to mitigate potential bias, the results need to be interpreted with caution. Finally, the study experienced a high dropout rate, which may have affected the quality of the study. To address this issue, we used several strategies to limit these problems. Reminders and follow-ups were sent to participants to encourage continued engagement. In addition, we included incentives for completing the study, such as double the amount of incentives and entry into a prize draw, to motivate participants to remain in the study. Despite these efforts, the high dropout rate remains a limitation, and future research should explore additional strategies to improve participant retention.

### Implications

This study provides evidence for the effectiveness of a WeChat-based intervention in improving influenza vaccine knowledge and attitudes among Chinese university students in the United Kingdom. The findings indicate that the cocreation method used has significant potential to engage this population effectively. The methods applied in this study offer valuable insights for future research evaluating social media interventions. The use of WeChat, combined with educational posts, personalized autoreplies, and timely reminders, demonstrated a successful approach to increasing health-related knowledge and fostering positive attitudes toward vaccination. Future studies can build on this framework to explore similar interventions in different contexts and populations, using social media to address various public health challenges. In practice, this intervention can be applied in collaboration with higher education agencies and student organizations. By embedding these functions within university health services and student support systems, institutions can enhance their efforts to promote community health. This approach can be particularly effective in reaching international students who may face barriers to accessing traditional health care resources. The success of this WeChat-based intervention highlights the potential of digital health strategies to address gaps in health care access and education. As social media continues to play an integral role in communication, its application in health promotion offers a promising avenue for public health initiatives.

### Conclusions

This study highlights the positive impact of a WeChat intervention on improving KAB related to SIV. It underscores the potential of digital health interventions to empower Chinese students with the knowledge to drive attitude and behavior change. Future longitudinal studies are needed to assess the long-term impact of such interventions on SIV rates and behaviors beyond the 4-week intervention period, and cross-cultural comparisons could provide valuable insights into the generalizability of our findings. Nevertheless, this research contributes to the growing body of literature on the effectiveness of digital health interventions and provides a foundation for future studies aiming to optimize similar interventions for various health contexts and populations.

## Appendix

Multimedia Appendix 1 Baseline and follow-up questionnaire.

Multimedia Appendix 2 Knowledge, attitude, behavior questions, answers, and coding details.

Multimedia Appendix 3 Statistics for between-group differences.

Multimedia Appendix 4 Statistics for users’ feedback.

## Abbreviations

CFI: comparative fit index
GFI: goodness of fit index
KAB: knowledge, attitude, and behavior
NFI: normed fit index
NNFI: nonnormed fit index
RMR: root mean square residual
RMSEA: root mean square error of approximation
SIV: seasonal influenza vaccination
