# Supplementary material for: Evaluating a WeChat-Based Intervention to Enhance Influenza Vaccination Knowledge, Attitude, and Behavior Among Chinese University Students Residing in the United Kingdom: Controlled, Quasi-Experimental, Mixed Methods Study
Source: JMIR Form Res. 2024 Oct 24;8:e55706. doi: 10.2196/55706 (PMC11544343; doi:10.2196/55706)
Supplement: Multimedia Appendix 1 [file formative_v8i1e55706_app1.pdf]

Baseline survey of influenza vaccine questionnaire [Baseline survey]

(All data you submit will be anonymised and encrypted, we will not pass on the information to any third party and the data will be used for research purposes only. This study has been approved by the University College London Ethics Review Board [study code:21647/001]. Before completing the questionnaire, please read the [Informed Consent Form]. If you agree to participate, please select consent and start answering.

- I agree to participate
- I refuse to participate

This is a study using pre- and post- quasi-experimental design. We would like to invite you to complete the survey again after **one month** (when you will be rewarded with a double bonus). Please provide your contact details preference: (Your contact details will be used for research only, will not be given to third parties and will be **permanently deleted** after the study, thank you for your participation!)

- **WeChat____**
- **Phone SMS ____**
- **Email ____**
- **QQ ____**
- **I don't want to participate the second survey**

  Your referrer is [optional]

- None
- Referrer number ____

======Page 2======

Section A. Basic information

1. Your year of birth is [drop down question]
2. Your gender is [one-selected]

   - Male
   - Female

3. The degree you are currently pursuing is [optional]

- Undergraduate
- Masters
- PhD

4. The city where your university is located is (in Chinese) [one-line text question] (e.g., London, Manchester, etc.)

5. Is your current major related to medical or healthcare [single choice]

   - Yes
   - No

6. How do you feel about your health? [Single question]

   - Very well
   - Well
   - Moderate
   - Bad
   - Very bad

======Page 3======
Section B. Health behaviour

1. Have you received the influenza vaccination since October 1st 2022

- Yes [Jump to last page]
- No [Jump to 2.]

2. How likely are you to receive the influenza vaccine within the next 3 months? [single question]

- Not at all
- Unlikely
- Not sure
- Likely
- Very likely

3. Do you have a regular annual flu vaccination? [Single question]

- Yes
- No

4. Have you taken the initiative to know about the flu vaccine, e.g. by search it online, consulting friends and doctors? [Single question]

- Yes
- No

5. The average amount of time you spend using WeChat each day is

- More than 3 hours
- 2-3 hours
- 1-2 hours
- Up to 1hour
- 0

6. How often do you read WeChat official accounts

- Multiple times a day
- Once a day
- At least once a week
- At least once a month
- Never

7. Have you ever learned about health-related information on WeChat official account? [Single-choice]

- Yes

- No

8. Have you ever **booked or had any vaccinations (e.g. COVID-19, HPV, flu, monkeypox vaccine, etc.) in the UK**? [Single question]

- Yes
- No

9. Have you ever **visited a GP or hospital in the UK**? [Single question]

- Yes
- No

10. Have you ever **caught a cold or flu in the UK**? [Single question]

- Yes
- No

11. How many doses of **COVID-19 vaccine** have you received? [Single question]

- 0 dose
- 1 dose
- 2 dose
- 3 dose
- 4 dose and above

12. Have you completed the full course of COVID-19 vaccination (excluding the booster)

- Yes
- No

13. Have you** got COVID-19 since arriving the UK**? [Single question]

- Yes, I have tested positive [continue with the question]
- No [skip to next page]
- I suspected I had it, but had no proof [skip to next page]
- Prefer not to disclose [skip to next page]

14. During your COVID-19 infection, how much do you think the following were affected [Matrix Scale: 1 to 5 No affect to severely affected]

o Physical health
o Mental Health
o Daily life
o Academic/work

15. What is the approximate number of days it took for you to recover? [Single choice] (You may fill in the numerical estimate based on your self-test)

- ____ days
- I'm not sure

======Page 4======

**C. Influenza vaccine knowledge assessment (the following influenza refers to seasonal influenza, not pandemic influenza) **
(5 questions for each of the following Learning objectives, one randomly selected for a total of ten questions per questionnaire)

Learning objective 1: At-risk group

The elderly, infants and young children and people with chronic illnesses are at high risk of influenza [Single-choice] (I think this statement is)

• Correct
• Wrong
• Not sure

Only older people are at high risk of influenza [single-choice] (I think this statement is) •

• Correct
• Wrong
• Not sure

Only infants and young children are at high risk for influenza [single-choice] (I think this statement is)

• Correct
• Wrong
• Not sure

Healthy adults are not at high risk for influenza [single choice] (I think this statement is)

• Correct
• Wrong
• Not sure

University students are at high risk of influenza [single-choice] (I think this statement is)

• Correct
• Wrong
• Not sure

Learning objective 2: Flu susceptibility

All people are generally susceptible to influenza [single choice] (I think this statement is)

- Correct
- Wrong
- Not sure

College students are generally susceptible to influenza [single-choice] (I think this statement is)

- Correct
- Wrong
- Not sure

Only the elderly, infants and young children and people with chronic illnesses are susceptible to influenza [single choice] (I think this statement is)

- Correct
- Wrong
- Not sure

Anyone who is generally susceptible to influenza [single choice] (I think this statement is)

- Correct
- Wrong
- Not sure

Only a small number of people are generally susceptible to influenza [single-choice] (I think this statement is)

- Correct
- Wrong
- Not sure

Learning objective 3: Flu symptoms

The main symptoms of influenza include cough, sore throat, runny nose and nasal congestion [single choice] (I think this statement is)

- Correct
- Wrong
- Not sure

The main symptoms of influenza include coughing and sore throat, nausea and vomiting, high fever and chills [Single-choice] (I think this statement is)

• Correct
• Wrong
• Not sure

The main symptoms of influenza include coughing and sore throat and gastrointestinal discomfort [single choice] (I think this statement is)

• Correct
• Wrong
• Not sure

Complications of influenza include pneumonia, encephalitis and myocarditis [Single-choice] (I think this statement is)

• Correct
• Wrong
• Not sure

Complications of influenza include pneumonia, meningitis and skin irritation [single choice] (I think this statement is)

• Correct
• Wrong
• Not sure

Learning objective 4: Timing of vaccination

In the UK, the best time to get a flu vaccination is between October and January [single-choice] (I think this statement is)

• Correct
• Wrong
• Not sure

In the UK, the best time to get a flu vaccination is between July and August [single choice] (I think this statement is)

• Correct
• Wrong
• Not sure

In the UK, the best time to get a flu vaccination is between January and April [single choice] (I think this statement is)

• Correct
• Wrong
• Not sure

In the UK, the best time to get a flu vaccination is in October [single choice] (I think this statement is)

• Correct
• Wrong
• Not sure

In the UK, the best season to get a flu vaccination is autumn and winter [single choice] (I think this statement is)

• Correct
• Wrong
• Not sure

Learning objective 5: Preventive measures for influenza

The preventive measures for influenza are similar to those for the COVID-19 [single choice] (I think this statement is)

• Correct
• Wrong
• Not sure

Wearing a mask and washing your hands regularly can prevent influenza [single choice] (I think this statement is)

• Correct
• Wrong
• Not sure

Influenza vaccination is the most effective means of preventing influenza [single choice] (I think this statement is)

• Correct
• Wrong
• Not sure

Flu vaccination does not prevent influenza [single-choice] (I think this statement is) • Correct

• Wrong
• Not sure

Flu shots can reduce the risk of serious illness from influenza [single choice] (I think this statement is)

• Correct
• Wrong
• Not sure

Learning objective 6: Flu severity

The number of hospitalizations due to influenza is about 3 to 5 million worldwide each year [single choice] (I think this statement is)

• Correct
• Wrong
• Not sure

The annual global death toll from influenza is about 2,00,000 to 5,00,000 [single-choice] (I think this statement is)

• Correct
• Wrong
• Not sure

The number of deaths due to influenza in China is about 88,000 each year [Single-choice] (I think this statement is)

• Correct
• Wrong
• Not sure

The general mortality rate for seasonal influenza is approximately 0.01% [single-choice] (I think this statement is)

• Correct
• Wrong
• Not sure

On average, about one person may die for every 10,000 cases of influenza [single choice] (I think this statement is)

• Correct
• Wrong
• Not sure

Learning objective 7: Where to get the vaccine

In the UK, the only way to get a flu vaccination is to go to hospital [single choice] (I think this statement is)

• Correct
• Wrong
• Not sure

In the UK, flu vaccination is only available at professional vaccination centres [single choice] (I think this statement is)

• Correct
• Wrong
• Not sure

The flu vaccine is also available in pharmacies in the UK [single choice] (I think this statement is)

• Correct
• Wrong
• Not sure

In the UK, you can only get the flu vaccine if you see a GP (General Practitioner) [single choice] (I think this statement is)

• Correct
• Wrong
• Not sure

In the UK, the flu vaccine is only available at NHS facilities [single-choice] (I think this statement is)

• Correct
• Wrong
• Not sure

Learning objective 8: Vaccination frequency

You need to get a flu vaccination once a year [single choice] (I think this statement is) • Correct

• Wrong
• Not sure

The flu vaccine is only needed once in a lifetime [single choice] (I think this statement is)

• Correct
• Wrong
• Not sure

I got the flu vaccination last year and I don't need it this year [single choice] (I think this statement is)

- Correct
- Wrong
- Not sure

Influenza vaccination is not required every year [single choice] (I think this statement is)

- Correct
- Wrong
- Not sure

You need to be vaccinated against influenza twice a year [single choice] (I think this statement is)

- Correct
- Wrong
- Not sure

Learning objective 9: Who need flu vaccination

Only people at high risk need to be vaccinated against influenza [single choice] (I think this statement is)

- Correct
- Wrong
- Not sure

Any susceptible person needs to be vaccinated against influenza [single choice] (I think this statement is)

- Correct
- Wrong
- Not sure

Anyone needs to be vaccinated against influenza [single choice] (I think this statement is)

- Correct
- Wrong
- Not sure

Anyone older than six months of age needs to be vaccinated against influenza [single choice] (I think this statement is)

- Correct
- Wrong
- Not sure

College students do not need to be vaccinated against influenza [single-choice] (I think this statement is)

• Correct
• Wrong
• Not sure

Learning objective 10: Flu vaccine benefits

You won't get the flu if you get the flu vaccine [single-choice] (I think this statement is)

• Correct
• Wrong
• Not sure

Influenza vaccination reduces the risk of catching influenza [single choice] (I think this statement is)

• Correct
• Wrong
• Not sure

Influenza vaccination reduces the risk of causing severe illness [single choice] (I think this statement is)

• Correct
• Wrong
• Not sure

Influenza vaccine protects for up to 6-8 months [single choice] (I think this statement is)

• Correct
• Wrong
• Not sure

The flu vaccine is not very effective in preventing influenza [single choice] (I think this statement is)

• Correct
• Wrong
• Not sure

Knowledge self-assessment

Please self-rate your knowledge of the following [Matrix scale questions 1 to 5]
o The symptoms of influenza
o Influenza prevention and treatment measures
o Influenza vaccine safety
o The role and effectiveness of influenza vaccines
o Who needs the flu vaccination
o How to book and get a flu vaccination in the UK

======Page 5======
D. Influenza vaccine attitude assessment [paragraph description]

Select your level of agreement with the following descriptions [Matrix Scale: 1 to 5 totally disagree to strongly agree]

- I believe the flu vaccine is safe.
- I believe the flu vaccine is effective.
- The flu vaccine is important for my health.
- I believe that the process of producing and administering influenza vaccines is safe and effective.
- The information promoted by the government, universities, and the NHS about the flu vaccine is trustworthy.
- If I don't get the flu shot, I may catch the flu.
- I think that the natural immunity that comes from having the flu is better than the flu shot.
- I think that the probability of me getting the flu is so low that I don't need the vaccine.
- I think even if I get the flu I can live with it, so I don't need the vaccination.
- The place and environment for receiving vaccinations in the UK is so bad that it makes me not want to get vaccinated.
- The processes of making appointments and receiving the flu vaccinations in the UK are easy to me and take little time.
- I believe I will be able to get the flu vaccine if needed.
- I can afford the flu vaccine cost.
- I always be proactive in keeping up with information about the flu vaccine.

What would you like to know about the flu vaccine? [Multiple choice].

- Necessity
- Safety
- Effectiveness
- Where to get the jab
- How to make appointments
- Considerations
- Nothing
- Other ____

Survey of influenza vaccine questionnaire [Review survey – Control group]

Have you subscribed to the WeChat official “XXXXX”?

- Yes, my nickname is______
- No

======Page 2======

**A. Influenza vaccine knowledge assessment (the following influenza refers to seasonal influenza, not pandemic influenza) **
(5 questions for each of the following Learning objectives, one randomly selected for a total of ten questions per questionnaire)

Learning objective 1: At-risk group

The elderly, infants and young children and people with chronic illnesses are at high risk of influenza [Single-choice] (I think this statement is)

• Correct
• Wrong
• Not sure

Only older people are at high risk of influenza [single-choice] (I think this statement is) •

• Correct
• Wrong
• Not sure

Only infants and young children are at high risk for influenza [single-choice] (I think this statement is)

• Correct
• Wrong
• Not sure

Healthy adults are not at high risk for influenza [single choice] (I think this statement is)

• Correct
• Wrong
• Not sure

University students are at high risk of influenza [single-choice] (I think this statement is)

• Correct
• Wrong
• Not sure

Learning objective 2: Flu susceptibility

All people are generally susceptible to influenza [single choice] (I think this statement is)

• Correct
• Wrong
• Not sure

College students are generally susceptible to influenza [single-choice] (I think this statement is)

• Correct
• Wrong
• Not sure

Only the elderly, infants and young children and people with chronic illnesses are susceptible to influenza [single choice] (I think this statement is)

• Correct
• Wrong
• Not sure

Anyone who is generally susceptible to influenza [single choice] (I think this statement is)

• Correct
• Wrong
• Not sure

Only a small number of people are generally susceptible to influenza [single-choice] (I think this statement is)

• Correct
• Wrong
• Not sure

Learning objective 3: Flu symptoms

The main symptoms of influenza include cough, sore throat, runny nose and nasal congestion [single choice] (I think this statement is)

• Correct
• Wrong
• Not sure

The main symptoms of influenza include coughing and sore throat, nausea and vomiting, high fever and chills [Single-choice] (I think this statement is)

- Correct
- Wrong
- Not sure

The main symptoms of influenza include coughing and sore throat and gastrointestinal discomfort [single choice] (I think this statement is)

- Correct
- Wrong
- Not sure

Complications of influenza include pneumonia, encephalitis and myocarditis [Single-choice] (I think this statement is)

- Correct
- Wrong
- Not sure

Complications of influenza include pneumonia, meningitis and skin irritation [single choice] (I think this statement is)

- Correct
- Wrong
- Not sure

Learning objective 4: Timing of vaccination

In the UK, the best time to get a flu vaccination is between October and January [single-choice] (I think this statement is)

- Correct
- Wrong
- Not sure

In the UK, the best time to get a flu vaccination is between July and August [single choice] (I think this statement is)

- Correct
- Wrong
- Not sure

In the UK, the best time to get a flu vaccination is between January and April [single choice] (I think this statement is)

• Correct
• Wrong
• Not sure

In the UK, the best time to get a flu vaccination is in October [single choice] (I think this statement is)

• Correct
• Wrong
• Not sure

In the UK, the best season to get a flu vaccination is autumn and winter [single choice] (I think this statement is)

• Correct
• Wrong
• Not sure

Learning objective 5: Preventive measures for influenza

The preventive measures for influenza are similar to those for the COVID-19 [single choice] (I think this statement is)

• Correct
• Wrong
• Not sure

Wearing a mask and washing your hands regularly can prevent influenza [single choice] (I think this statement is)

• Correct
• Wrong
• Not sure

Influenza vaccination is the most effective means of preventing influenza [single choice] (I think this statement is)

• Correct
• Wrong
• Not sure

Flu vaccination does not prevent influenza [single-choice] (I think this statement is) • Correct

• Wrong
• Not sure

Flu shots can reduce the risk of serious illness from influenza [single choice] (I think this statement is)

• Correct
• Wrong
• Not sure

Learning objective 6: Flu severity

The number of hospitalizations due to influenza is about 3 to 5 million worldwide each year [single choice] (I think this statement is)

• Correct
• Wrong
• Not sure

The annual global death toll from influenza is about 2,00,000 to 5,00,000 [single-choice] (I think this statement is)

• Correct
• Wrong
• Not sure

The number of deaths due to influenza in China is about 88,000 each year [Single-choice] (I think this statement is)

• Correct
• Wrong
• Not sure

The general mortality rate for seasonal influenza is approximately 0.01% [single-choice] (I think this statement is)

• Correct
• Wrong
• Not sure

On average, about one person may die for every 10,000 cases of influenza [single choice] (I think this statement is)

• Correct
• Wrong
• Not sure

Learning objective 7: Where to get the vaccine

In the UK, the only way to get a flu vaccination is to go to hospital [single choice] (I think this statement is)

- Correct
- Wrong
- Not sure

In the UK, flu vaccination is only available at professional vaccination centres [single choice] (I think this statement is)

- Correct
- Wrong
- Not sure

The flu vaccine is also available in pharmacies in the UK [single choice] (I think this statement is)

- Correct
- Wrong
- Not sure

In the UK, you can only get the flu vaccine if you see a GP (General Practitioner) [single choice] (I think this statement is)

- Correct
- Wrong
- Not sure

In the UK, the flu vaccine is only available at NHS facilities [single-choice] (I think this statement is)

- Correct
- Wrong
- Not sure

Learning objective 8: Vaccination frequency

You need to get a flu vaccination once a year [single choice] (I think this statement is) • Correct

- Wrong
- Not sure

The flu vaccine is only needed once in a lifetime [single choice] (I think this statement is)

- Correct
- Wrong
- Not sure

I got the flu vaccination last year and I don't need it this year [single choice] (I think this statement is)

- Correct
- Wrong
- Not sure

Influenza vaccination is not required every year [single choice] (I think this statement is)

- Correct
- Wrong
- Not sure

You need to be vaccinated against influenza twice a year [single choice] (I think this statement is)

- Correct
- Wrong
- Not sure

Learning objective 9: Who need flu vaccination

Only people at high risk need to be vaccinated against influenza [single choice] (I think this statement is)

- Correct
- Wrong
- Not sure

Any susceptible person needs to be vaccinated against influenza [single choice] (I think this statement is)

- Correct
- Wrong
- Not sure

Anyone needs to be vaccinated against influenza [single choice] (I think this statement is)

- Correct
- Wrong
- Not sure

Anyone older than six months of age needs to be vaccinated against influenza [single choice] (I think this statement is)

- Correct
- Wrong
- Not sure

College students do not need to be vaccinated against influenza [single-choice] (I think this statement is)

• Correct
• Wrong
• Not sure

Learning objective 10: Flu vaccine benefits

You won't get the flu if you get the flu vaccine [single-choice] (I think this statement is)

• Correct
• Wrong
• Not sure

Influenza vaccination reduces the risk of catching influenza [single choice] (I think this statement is)

• Correct
• Wrong
• Not sure

Influenza vaccination reduces the risk of causing severe illness [single choice] (I think this statement is)

• Correct
• Wrong
• Not sure

Influenza vaccine protects for up to 6-8 months [single choice] (I think this statement is)

• Correct
• Wrong
• Not sure

The flu vaccine is not very effective in preventing influenza [single choice] (I think this statement is)

• Correct
• Wrong
• Not sure

Knowledge self-assessment

Please self-rate your knowledge of the following [Matrix scale questions 1 to 5]
o The symptoms of influenza
o Influenza prevention and treatment measures
o Influenza vaccine safety
o The role and effectiveness of influenza vaccines
o Who needs the flu vaccination
o How to book and get a flu vaccination in the UK

======Page 3======
D. Influenza vaccine attitude assessment [paragraph description]

Select your level of agreement with the following descriptions [Matrix Scale: 1 to 5 totally disagree to strongly agree]

- o I believe the flu vaccine is safe.
- o I believe the flu vaccine is effective.
- o The flu vaccine is important for my health.
- o I believe that the process of producing and administering influenza vaccines is safe and effective.
- o The information promoted by the government, universities, and the NHS about the flu vaccine is trustworthy.
- o If I don't get the flu shot, I may catch the flu.
- o I think that the natural immunity that comes from having the flu is better than the flu shot.
- o I think that the probability of me getting the flu is so low that I don't need the vaccine.
- o I think even if I get the flu I can live with it, so I don't need the vaccination.
- o The place and environment for receiving vaccinations in the UK is so bad that it makes me not want to get vaccinated.
- o The processes of making appointments and receiving the flu vaccinations in the UK are easy to me and take little time.
- o I believe I will be able to get the flu vaccine if needed.
- o I can afford the flu vaccine cost.
- o I always be proactive in keeping up with information about the flu vaccine.

What would you like to know about the flu vaccine? [Multiple choice].

- Necessity
- Safety
- Effectiveness
- Where to get the jab
- How to make appointments
- Considerations
- Nothing
- Other ____

======Page 3======
B. Influenza vaccine attitude assessment [paragraph description]

Select your level of agreement with the following descriptions [Matrix Scale: 1 to 5 totally disagree to strongly agree]

- o I believe the flu vaccine is safe.
- o I believe the flu vaccine is effective.
- o The flu vaccine is important for my health.
- o I believe that the process of producing and administering influenza vaccines is safe and effective.

- The information promoted by the government, universities, and the NHS about the flu vaccine is trustworthy.
- If I don't get the flu shot, I may catch the flu.
- I think that the natural immunity that comes from having the flu is better than the flu shot.
- I think that the probability of me getting the flu is so low that I don't need the vaccine.
- I think even if I get the flu I can live with it, so I don't need the vaccination.
- The place and environment for receiving vaccinations in the UK is so bad that it makes me not want to get vaccinated.
- The processes of making appointments and receiving the flu vaccinations in the UK are easy for me and take little time.
- I believe I will be able to get the flu vaccine if needed.
- I can afford the flu vaccine cost.
- I always be proactive in keeping up with information about the flu vaccine.

======Page 4======

D. Health Behaviors [paragraph description] [optional]

Since you answered the last questionnaire to date, have you received a flu vaccine [Single-Choice Question] [Required Answer]

- Vaccinated
- Not yet vaccinated, but made an appointment
- No vaccination plan

How likely are you to receive the influenza vaccine within the next 3 months? [Single-Choice Question] (If you have already received the vaccination, please skip this question)

- Not at all
- Unlikely
- Not sure
- Likely
- Very likely

Since the last time you completed the questionnaire, have you had a cold, COVID-19 or flu? [Single-choice question][Required answer]

- Yes
- No

Since the last time you filled out the questionnaire, have you learned about the flu vaccine through other channels, such as internet search, consulting your doctor, or friend, etc.? [Single Choice] [Mandatory Answer]

- Yes

- No

Have you read the articles published by “XXXXX”? [Single-choice question][Required Answer]

- Yes
- No

What is the most impressive article in your mind? [Single-response question][Optional] (If you have not read the article, please skip it.)

The channel through which you came into the official account “XXXXX”? is [Single-line text question][Optional answer](Please skip if you have not viewed it.)

- Recommended by friends
- Swiped by friends
- Group recommendation
- WeChat Search
- Other ____

In order to find out more about your review, we will be conducting telephone interviews with our users in the coming weeks, would you like to be interviewed back by phone (30 minutes)? You will be paid 5-10 pounds in cash. [Single Choice] [Mandatory].

- I do, I can be reached at ____
- No

Do you have any other suggestions for us (or this topic)? [multi-line text question] [optional answer]

Survey of influenza vaccine questionnaire [Review survey – Intervention group]

Have you subscribed to the WeChat official "XXXXX"?

- Yes, my nickname is______
- No

======Page 2======

**A. Influenza vaccine knowledge assessment (the following influenza refers to seasonal influenza, not pandemic influenza) **
(5 questions for each of the following Learning objectives, one randomly selected for a total of ten questions per questionnaire)

Learning objective 1: At-risk group

The elderly, infants and young children and people with chronic illnesses are at high risk of influenza [Single-choice] (I think this statement is)

• Correct
• Wrong
• Not sure

Only older people are at high risk of influenza [single-choice] (I think this statement is) •

• Correct
• Wrong
• Not sure

Only infants and young children are at high risk for influenza [single-choice] (I think this statement is)

• Correct
• Wrong
• Not sure

Healthy adults are not at high risk for influenza [single choice] (I think this statement is)

• Correct
• Wrong
• Not sure

University students are at high risk of influenza [single-choice] (I think this statement is)

• Correct
• Wrong
• Not sure

Learning objective 2: Flu susceptibility

All people are generally susceptible to influenza [single choice] (I think this statement is)

• Correct
• Wrong
• Not sure

College students are generally susceptible to influenza [single-choice] (I think this statement is)

• Correct
• Wrong
• Not sure

Only the elderly, infants and young children and people with chronic illnesses are susceptible to influenza [single choice] (I think this statement is)

• Correct
• Wrong
• Not sure

Anyone who is generally susceptible to influenza [single choice] (I think this statement is)

• Correct
• Wrong
• Not sure

Only a small number of people are generally susceptible to influenza [single-choice] (I think this statement is)

• Correct
• Wrong
• Not sure

Learning objective 3: Flu symptoms

The main symptoms of influenza include cough, sore throat, runny nose and nasal congestion [single choice] (I think this statement is)

• Correct
• Wrong
• Not sure

The main symptoms of influenza include coughing and sore throat, nausea and vomiting, high fever and chills [Single-choice] (I think this statement is)

• Correct
• Wrong
• Not sure

The main symptoms of influenza include coughing and sore throat and gastrointestinal discomfort [single choice] (I think this statement is)

• Correct
• Wrong
• Not sure

Complications of influenza include pneumonia, encephalitis and myocarditis [Single-choice] (I think this statement is)

• Correct
• Wrong
• Not sure

Complications of influenza include pneumonia, meningitis and skin irritation [single choice] (I think this statement is)

• Correct
• Wrong
• Not sure

Learning objective 4: Timing of vaccination

In the UK, the best time to get a flu vaccination is between October and January [single-choice] (I think this statement is)

• Correct
• Wrong
• Not sure

In the UK, the best time to get a flu vaccination is between July and August [single choice] (I think this statement is)

• Correct
• Wrong
• Not sure

In the UK, the best time to get a flu vaccination is between January and April [single choice] (I think this statement is)

• Correct
• Wrong
• Not sure

In the UK, the best time to get a flu vaccination is in October [single choice] (I think this statement is)

• Correct
• Wrong
• Not sure

In the UK, the best season to get a flu vaccination is autumn and winter [single choice] (I think this statement is)

• Correct
• Wrong
• Not sure

Learning objective 5: Preventive measures for influenza

The preventive measures for influenza are similar to those for the COVID-19 [single choice] (I think this statement is)

• Correct
• Wrong
• Not sure

Wearing a mask and washing your hands regularly can prevent influenza [single choice] (I think this statement is)

• Correct
• Wrong
• Not sure

Influenza vaccination is the most effective means of preventing influenza [single choice] (I think this statement is)

• Correct
• Wrong
• Not sure

Flu vaccination does not prevent influenza [single-choice] (I think this statement is) • Correct

• Wrong
• Not sure

Flu shots can reduce the risk of serious illness from influenza [single choice] (I think this statement is)

• Correct
• Wrong
• Not sure

Learning objective 6: Flu severity

The number of hospitalizations due to influenza is about 3 to 5 million worldwide each year [single choice] (I think this statement is)

• Correct
• Wrong
• Not sure

The annual global death toll from influenza is about 2,00,000 to 5,00,000 [single-choice] (I think this statement is)

• Correct
• Wrong
• Not sure

The number of deaths due to influenza in China is about 88,000 each year [Single-choice] (I think this statement is)

• Correct
• Wrong
• Not sure

The general mortality rate for seasonal influenza is approximately 0.01% [single-choice] (I think this statement is)

• Correct
• Wrong
• Not sure

On average, about one person may die for every 10,000 cases of influenza [single choice] (I think this statement is)

• Correct
• Wrong
• Not sure

Learning objective 7: Where to get the vaccine

In the UK, the only way to get a flu vaccination is to go to hospital [single choice] (I think this statement is)

- Correct
- Wrong
- Not sure

In the UK, flu vaccination is only available at professional vaccination centres [single choice] (I think this statement is)

- Correct
- Wrong
- Not sure

The flu vaccine is also available in pharmacies in the UK [single choice] (I think this statement is)

- Correct
- Wrong
- Not sure

In the UK, you can only get the flu vaccine if you see a GP (General Practitioner) [single choice] (I think this statement is)

- Correct
- Wrong
- Not sure

In the UK, the flu vaccine is only available at NHS facilities [single-choice] (I think this statement is)

- Correct
- Wrong
- Not sure

Learning objective 8: Vaccination frequency

You need to get a flu vaccination once a year [single choice] (I think this statement is) • Correct

- Wrong
- Not sure

The flu vaccine is only needed once in a lifetime [single choice] (I think this statement is)

- Correct
- Wrong
- Not sure

I got the flu vaccination last year and I don't need it this year [single choice] (I think this statement is)

- Correct
- Wrong
- Not sure

Influenza vaccination is not required every year [single choice] (I think this statement is)

- Correct
- Wrong
- Not sure

You need to be vaccinated against influenza twice a year [single choice] (I think this statement is)

- Correct
- Wrong
- Not sure

Learning objective 9: Who need flu vaccination

Only people at high risk need to be vaccinated against influenza [single choice] (I think this statement is)

- Correct
- Wrong
- Not sure

Any susceptible person needs to be vaccinated against influenza [single choice] (I think this statement is)

- Correct
- Wrong
- Not sure

Anyone needs to be vaccinated against influenza [single choice] (I think this statement is)

- Correct
- Wrong
- Not sure

Anyone older than six months of age needs to be vaccinated against influenza [single choice] (I think this statement is)

- Correct
- Wrong
- Not sure

College students do not need to be vaccinated against influenza [single-choice] (I think this statement is)

• Correct
• Wrong
• Not sure

Learning objective 10: Flu vaccine benefits

You won't get the flu if you get the flu vaccine [single-choice] (I think this statement is)

• Correct
• Wrong
• Not sure

Influenza vaccination reduces the risk of catching influenza [single choice] (I think this statement is)

• Correct
• Wrong
• Not sure

Influenza vaccination reduces the risk of causing severe illness [single choice] (I think this statement is)

• Correct
• Wrong
• Not sure

Influenza vaccine protects for up to 6-8 months [single choice] (I think this statement is)

• Correct
• Wrong
• Not sure

The flu vaccine is not very effective in preventing influenza [single choice] (I think this statement is)

• Correct
• Wrong
• Not sure

Knowledge self-assessment

Please self-rate your knowledge of the following [Matrix scale questions 1 to 5]
o The symptoms of influenza
o Influenza prevention and treatment measures
o Influenza vaccine safety
o The role and effectiveness of influenza vaccines
o Who needs the flu vaccination
o How to book and get a flu vaccination in the UK

======Page 3======
B. Influenza vaccine attitude assessment [paragraph description]

Select your level of agreement with the following descriptions [Matrix Scale: 1 to 5 totally disagree to strongly agree]

- I believe the flu vaccine is safe.
- I believe the flu vaccine is effective.
- The flu vaccine is important for my health.
- I believe that the process of producing and administering influenza vaccines is safe and effective.
- The information promoted by the government, universities, and the NHS about the flu vaccine is trustworthy.
- If I don't get the flu shot, I may catch the flu.
- I think that the natural immunity that comes from having the flu is better than the flu shot.
- I think that the probability of me getting the flu is so low that I don't need the vaccine.
- I think even if I get the flu I can live with it, so I don't need the vaccination.
- The place and environment for receiving vaccinations in the UK is so bad that it makes me not want to get vaccinated.
- The processes of making appointments and receiving the flu vaccinations in the UK are easy for me and take little time.
- I believe I will be able to get the flu vaccine if needed.
- I can afford the flu vaccine cost.
- I always be proactive in keeping up with information about the flu vaccine.

======Page 4======

D. Health Behaviors [paragraph description] [optional]

Since you answered the last questionnaire to date, have you received a flu vaccine [Single-Choice Question] [Required Answer]

- Vaccinated
- Not yet vaccinated, but made an appointment
- No vaccination plan

How likely are you to receive the influenza vaccine within the next 3 months? [Single-Choice Question] (If you have already received the vaccination, please skip this question)

- Not at all
- Unlikely
- Not sure
- Likely
- Very likely

Since the last time you completed the questionnaire, have you had a cold, COVID-19 or flu? [Single-choice question][Required answer]

- Yes
- No

Since the last time you filled out the questionnaire, have you taken the initiative to learn about the flu vaccine through other channels, such as internet searching, consulting your doctor, or friends, etc.? [Single-Choice Question] [Required Answer]

- Yes
- No

===**Page 5**===

Evaluation of the use of the official account [paragraph description][optional]

What functions have you used in the "XXXXX" official account? [Multiple choice][Required answer]

- Read article
- Reply with keywords to get appointment information
- Forwarding information
- Fill in the questionnaire
- Comment or ask a question
- None of them have been used
- Other ____

How often do you check the official account?[Single choice question][Required answer]

- Multiple times per day
- Once a day
- At least once a week
- At least once a month
- Only when needed
- Never

What impressed you most about the official account articles [one-line text question][mandatory answer] (please describe the article or provide keywords)

How helpful do you think this official account has been in keeping you informed about the flu vaccine? [Single line text question] [Required Answer]

- Extremely
- Fairly
- Average
- Comparatively small
- Very small

How helpful do you think this official account would be in helping you choose whether or not to get a flu shot? [Single Choice Question] [Required Answer]

- Extremely
- Comparatively large
- Average
- Comparatively small
- Extremely small

Please rate your level of agreement with the following statements [Matrix Scale Questions] [Mandatory] (5 marks indicate strong agreement, 1 mark indicates strong disagreement, the lower the score, the lower the level of agreement)
1~5

1.I'm willing to continue following and using it
2.It is easy to use.
3.Its content and features are appealing.
4.Its content and features align with my needs
5.The contents are interesting.
6.The information provided are trustworthy
7.It is useful.
8.I am willing to recommend it or its content to my friends
9.It is helpful in providing SIV information
10.It is helpful in making SIV decisions

In order to find out more about your experience and comments, we will be conducting telephone interviews with our users in the coming weeks, would you be willing to have a call back (30 minutes)? You will be paid 5-10 pounds in cash. [Single Choice Question] [Mandatory Answer]

- I do, I can be reached at ____
- No, I don't want to.

What is your opinion of the official account? [Multi-line text question] [Optional answer] (e.g., would you like to continue using this official account, please state why)

Do you have any other suggestions for us (or this topic)? [Multi-line text question] [optional answer]
