# Supplementary material for: Evaluating a WeChat-Based Intervention to Enhance Influenza Vaccination Knowledge, Attitude, and Behavior Among Chinese University Students Residing in the United Kingdom: Controlled, Quasi-Experimental, Mixed Methods Study
Source: JMIR Form Res. 2024 Oct 24;8:e55706. doi: 10.2196/55706 (PMC11544343; doi:10.2196/55706)
Supplement: Multimedia Appendix 2 [file formative_v8i1e55706_app2.pdf]

KAB scores & coding details

```
########## unsubscriber - pre
un_pre <- read.csv(file = "control_cleaned.csv", header = TRUE)
back_up<-un_pre

## calculate knowldege scores – control group

for (i in 1:293) {
if(!is.na(un_pre$K1_1[i] == 1 | un_pre$K1_2[i] == 2| un_pre$K1_3[i] == 2| un_pre$K1_4[i]
== 1| un_pre$K1_5[i] == 1)){
  un_pre$K1[i] <- "CORRECT"
} else {
  un_pre$K1[i] <- "WRONG"
}
}

for (i in 1:293) {
  if(!is.na(un_pre$K2_1[i] == 1 | un_pre$K2_2[i] == 1| un_pre$K2_3[i] == 2| un_pre$K2_4[i]
== 1| un_pre$K2_5[i] == 2)){
    un_pre$K2[i] <- "CORRECT"
  } else {
    un_pre$K2[i] <- "WRONG"
  }
}

for (i in 1:293) {
  if(!is.na(un_pre$K3_1[i] == 1 | un_pre$K3_2[i] == 1| un_pre$K3_3[i] == 2| un_pre$K3_4[i]
== 1| un_pre$K3_5[i] == 2)){
    un_pre$K3[i] <- "CORRECT"
  } else {
    un_pre$K3[i] <- "WRONG"
  }
}

for (i in 1:293) {
  if(!is.na(un_pre$K4_1[i] == 1 | un_pre$K4_2[i] == 2| un_pre$K4_3[i] == 2| un_pre$K4_4[i]
== 1| un_pre$K4_5[i] == 1)){
    un_pre$K4[i] <- "CORRECT"
  } else {
    un_pre$K4[i] <- "WRONG"
  }
}

for (i in 1:293) {
```

```
  if(!is.na(un_pre$K5_1[i] == 1 | un_pre$K5_2[i] == 1| un_pre$K5_3[i] == 1| un_pre$K5_4[i]
== 2| un_pre$K5_5[i] == 1)){
    un_pre$K5[i] <- "CORRECT"
  } else {
    un_pre$K5[i] <- "WRONG"
  }
}

for (i in 1:293) {
  if(!is.na(un_pre$K6_1[i] == 1 | un_pre$K6_2[i] == 2| un_pre$K6_3[i] == 1| un_pre$K6_4[i]
== 2| un_pre$K6_5[i] == 2)){
    un_pre$K6[i] <- "CORRECT"
  } else {
    un_pre$K6[i] <- "WRONG"
  }
}

for (i in 1:293) {
  if(!is.na(un_pre$K7_1[i] == 2 | un_pre$K7_2[i] == 2| un_pre$K7_3[i] == 1| un_pre$K7_4[i]
== 2| un_pre$K7_5[i] == 2)){
    un_pre$K7[i] <- "CORRECT"
  } else {
    un_pre$K7[i] <- "WRONG"
  }
}

for (i in 1:293) {
  if(!is.na(un_pre$K8_1[i] == 1 | un_pre$K8_2[i] == 2| un_pre$K8_3[i] == 2| un_pre$K8_4[i]
== 2| un_pre$K8_5[i] == 2)){
    un_pre$K8[i] <- "CORRECT"
  } else {
    un_pre$K8[i] <- "WRONG"
  }
}

for (i in 1:293) {
  if(!is.na(un_pre$K9_1[i] == 2 | un_pre$K9_2[i] == 1| un_pre$K9_3[i] == 2| un_pre$K9_4[i]
== 1| un_pre$K9_5[i] == 2)){
    un_pre$K9[i] <- "CORRECT"
  } else {
    un_pre$K9[i] <- "WRONG"
  }
}

for (i in 1:293) {
  if(!is.na(un_pre$K10_1[i] == 2 | un_pre$K10_2[i] == 1| un_pre$K10_3[i] == 1|
un_pre$K10_4[i] == 1| un_pre$K10_5[i] == 2)){
```

```
    un_pre$K10[i] <- "CORRECT"
  } else {
    un_pre$K10[i] <- "WRONG"
  }
}

# assign scores for answers
un_pre[un_pre == "CORRECT"] <- 1
un_pre[un_pre == "WRONG"] <- 0

# change data type for calculation
for (i in 108:117){
  un_pre[,i] <- as.numeric(un_pre[,i])
}

# calculate sum scores
un_pre$K_SCORE <- rowSums(un_pre[, 108:117])
mean(un_pre$K_SCORE)
sd(un_pre$K_SCORE)

# calculate self-reported knowledge scores
un_pre$K_SELF_mean <- rowSums(un_pre[, 79:84])/6
mean(un_pre$K_SELF_mean)
sd(un_pre$K_SELF_mean)

# calculate statistics
un_pre_result <- c(mean(un_pre$K_SCORE),sd(un_pre$K_SCORE),
mean(un_pre$K_SELF_mean),sd(un_pre$K_SELF_mean) )

#### attitude score
# reverse coding
un_pre$A7 = 6-un_pre$A7
un_pre$A8 = 6-un_pre$A8
un_pre$A9 = 6-un_pre$A9
un_pre$A10 = 6-un_pre$A10

# calculate mean score & statistics
un_pre$A_mean <- rowSums(un_pre[, 85:98])/14
mean(un_pre$A_mean)
sd(un_pre$A_mean)

# collate result
un_pre_result <- c(mean(un_pre$K_SCORE),sd(un_pre$K_SCORE),
mean(un_pre$K_SELF_mean),sd(un_pre$K_SELF_mean) , mean(un_pre$A_mean),
sd(un_pre$A_mean))
result <- data.frame(un_pre_result)
result <- t(result)
```

```
names <- c("K_mean", "K_sd", "K_self_mean", "K_self_sd", "A_mean", "A_sd")
colnames(result) <- names

########### intervention group - pre####################################

sub_pre <- read.csv(file = "sub_cleanned.csv", header = TRUE)
back_up_sub<-sub_pre
sub_pre <- sub_pre[-c(205,212),]

## calculate knowldege scores

for (i in 1:303) {
  if(!is.na(sub_pre$K1_1[i] == 1 | sub_pre$K1_2[i] == 2| sub_pre$K1_3[i] == 2|
sub_pre$K1_4[i] == 1| sub_pre$K1_5[i] == 1)){
    sub_pre$K1[i] <- "CORRECT"
  } else {
    sub_pre$K1[i] <- "WRONG"
  }
}

sub_pre_test <- sub_pre[,c("K1_1", "K1_2", "K1_3","K1_4","K1_5","K1")]

for (i in 1:303) {
  if(!is.na(sub_pre$K2_1[i] == 1 | sub_pre$K2_2[i] == 1| sub_pre$K2_3[i] == 2|
sub_pre$K2_4[i] == 1| sub_pre$K2_5[i] == 2)){
    sub_pre$K2[i] <- "CORRECT"
  } else {
    sub_pre$K2[i] <- "WRONG"
  }
}

for (i in 1:303) {
  if(!is.na(sub_pre$K3_1[i] == 1 | sub_pre$K3_2[i] == 1| sub_pre$K3_3[i] == 2|
sub_pre$K3_4[i] == 1| sub_pre$K3_5[i] == 2)){
    sub_pre$K3[i] <- "CORRECT"
  } else {
    sub_pre$K3[i] <- "WRONG"
  }
}

for (i in 1:303) {
  if(!is.na(sub_pre$K4_1[i] == 1 | sub_pre$K4_2[i] == 2| sub_pre$K4_3[i] == 2|
sub_pre$K4_4[i] == 1| sub_pre$K4_5[i] == 1)){
    sub_pre$K4[i] <- "CORRECT"
  } else {
    sub_pre$K4[i] <- "WRONG"
  }
```

```
}

for (i in 1:303) {
  if(!is.na(sub_pre$K5_1[i] == 1 | sub_pre$K5_2[i] == 1| sub_pre$K5_3[i] == 1|
sub_pre$K5_4[i] == 2| sub_pre$K5_5[i] == 1)){
    sub_pre$K5[i] <- "CORRECT"
  } else {
    sub_pre$K5[i] <- "WRONG"
  }
}

for (i in 1:303) {
  if(!is.na(sub_pre$K6_1[i] == 1 | sub_pre$K6_2[i] == 2| sub_pre$K6_3[i] == 1|
sub_pre$K6_4[i] == 2| sub_pre$K6_5[i] == 2)){
    sub_pre$K6[i] <- "CORRECT"
  } else {
    sub_pre$K6[i] <- "WRONG"
  }
}

for (i in 1:303) {
  if(!is.na(sub_pre$K7_1[i] == 2 | sub_pre$K7_2[i] == 2| sub_pre$K7_3[i] == 1|
sub_pre$K7_4[i] == 2| sub_pre$K7_5[i] == 2)){
    sub_pre$K7[i] <- "CORRECT"
  } else {
    sub_pre$K7[i] <- "WRONG"
  }
}

for (i in 1:303) {
  if(!is.na(sub_pre$K8_1[i] == 1 | sub_pre$K8_2[i] == 2| sub_pre$K8_3[i] == 2|
sub_pre$K8_4[i] == 2| sub_pre$K8_5[i] == 2)){
    sub_pre$K8[i] <- "CORRECT"
  } else {
    sub_pre$K8[i] <- "WRONG"
  }
}

for (i in 1:303) {
  if(!is.na(sub_pre$K9_1[i] == 2 | sub_pre$K9_2[i] == 1| sub_pre$K9_3[i] == 2|
sub_pre$K9_4[i] == 1| sub_pre$K9_5[i] == 2)){
    sub_pre$K9[i] <- "CORRECT"
  } else {
    sub_pre$K9[i] <- "WRONG"
  }
}
```

```
for (i in 1:303) {
  if(!is.na(sub_pre$K10_1[i] == 2 | sub_pre$K10_2[i] == 1| sub_pre$K10_3[i] == 1|
sub_pre$K10_4[i] == 1| sub_pre$K10_5[i] == 2)){
    sub_pre$K10[i] <- "CORRECT"
  } else {
    sub_pre$K10[i] <- "WRONG"
  }
}

sub_pre[sub_pre == "CORRECT"] <- 1
sub_pre[sub_pre == "WRONG"] <- 0

for (i in 108:117){
  sub_pre[,i] <- as.numeric(sub_pre[,i])
}

sub_pre$K_SCORE <- rowSums(sub_pre[, 108:117])
mean(sub_pre$K_SCORE)
sd(sub_pre$K_SCORE)

sub_pre$K_SELF_mean <- rowSums(sub_pre[, 79:84])/6
mean(sub_pre$K_SELF_mean)
sd(sub_pre$K_SELF_mean)


## attitude
sub_pre$A7 = 6-sub_pre$A7
sub_pre$A8 = 6-sub_pre$A8
sub_pre$A9 = 6-sub_pre$A9
sub_pre$A10 = 6-sub_pre$A10

sub_pre$A_mean <- rowSums(sub_pre[, 85:98])/14
mean(sub_pre$A_mean)
sd(sub_pre$A_mean)

## collate result
sub_pre_result <- c(mean(sub_pre$K_SCORE),sd(sub_pre$K_SCORE),
mean(sub_pre$K_SELF_mean),sd(sub_pre$K_SELF_mean) , mean(sub_pre$A_mean),
sd(sub_pre$A_mean))
result <- data.frame(un_pre_result, sub_pre_result)
result <- t(result)
names <- c("K_mean", "K_sd", "K_self_mean", "K_self_sd", "A_mean", "A_sd")
colnames(result) <- names


################## intervention - post ######################
```

```
sub_post <- read.csv(file = "Subscribers_cleaned.csv", header = TRUE)
back_up_sub_post<-sub_post

## calculate knowldege scores

for (i in 1:22) {
  if(!is.na(sub_post$K1_1[i] == 1 | sub_post$K1_2[i] == 2| sub_post$K1_3[i] == 2|
sub_post$K1_4[i] == 1| sub_post$K1_5[i] == 1)){
    sub_post$K1[i] <- "CORRECT"
  } else {
    sub_post$K1[i] <- "WRONG"
  }
}

sub_post_test <- sub_post[,c("K1_1", "K1_2", "K1_3","K1_4","K1_5","K1")]

for (i in 1:22) {
  if(!is.na(sub_post$K2_1[i] == 1 | sub_post$K2_2[i] == 1| sub_post$K2_3[i] == 2|
sub_post$K2_4[i] == 1| sub_post$K2_5[i] == 2)){
    sub_post$K2[i] <- "CORRECT"
  } else {
    sub_post$K2[i] <- "WRONG"
  }
}

for (i in 1:22) {
  if(!is.na(sub_post$K3_1[i] == 1 | sub_post$K3_2[i] == 1| sub_post$K3_3[i] == 2|
sub_post$K3_4[i] == 1| sub_post$K3_5[i] == 2)){
    sub_post$K3[i] <- "CORRECT"
  } else {
    sub_post$K3[i] <- "WRONG"
  }
}

for (i in 1:22) {
  if(!is.na(sub_post$K4_1[i] == 1 | sub_post$K4_2[i] == 2| sub_post$K4_3[i] == 2|
sub_post$K4_4[i] == 1| sub_post$K4_5[i] == 1)){
    sub_post$K4[i] <- "CORRECT"
  } else {
    sub_post$K4[i] <- "WRONG"
  }
}

for (i in 1:22) {
  if(!is.na(sub_post$K5_1[i] == 1 | sub_post$K5_2[i] == 1| sub_post$K5_3[i] == 1|
sub_post$K5_4[i] == 2| sub_post$K5_5[i] == 1)){
    sub_post$K5[i] <- "CORRECT"
```

```
  } else {
    sub_post$K5[i] <- "WRONG"
  }
}

for (i in 1:22) {
  if(!is.na(sub_post$K6_1[i] == 1 | sub_post$K6_2[i] == 2| sub_post$K6_3[i] == 1|
sub_post$K6_4[i] == 2| sub_post$K6_5[i] == 2)){
    sub_post$K6[i] <- "CORRECT"
  } else {
    sub_post$K6[i] <- "WRONG"
  }
}

for (i in 1:22) {
  if(!is.na(sub_post$K7_1[i] == 2 | sub_post$K7_2[i] == 2| sub_post$K7_3[i] == 1|
sub_post$K7_4[i] == 2| sub_post$K7_5[i] == 2)){
    sub_post$K7[i] <- "CORRECT"
  } else {
    sub_post$K7[i] <- "WRONG"
  }
}

for (i in 1:22) {
  if(!is.na(sub_post$K8_1[i] == 1 | sub_post$K8_2[i] == 2| sub_post$K8_3[i] == 2|
sub_post$K8_4[i] == 2| sub_post$K8_5[i] == 2)){
    sub_post$K8[i] <- "CORRECT"
  } else {
    sub_post$K8[i] <- "WRONG"
  }
}

for (i in 1:22) {
  if(!is.na(sub_post$K9_1[i] == 2 | sub_post$K9_2[i] == 1| sub_post$K9_3[i] == 2|
sub_post$K9_4[i] == 1| sub_post$K9_5[i] == 2)){
    sub_post$K9[i] <- "CORRECT"
  } else {
    sub_post$K9[i] <- "WRONG"
  }
}

for (i in 1:22) {
  if(!is.na(sub_post$K10_1[i] == 2 | sub_post$K10_2[i] == 1| sub_post$K10_3[i] == 1|
sub_post$K10_4[i] == 1| sub_post$K10_5[i] == 2)){
    sub_post$K10[i] <- "CORRECT"
  } else {
    sub_post$K10[i] <- "WRONG"
```

```
  }
}

sub_post[sub_post == "CORRECT"] <- 1
sub_post[sub_post == "WRONG"] <- 0

for (i in 105:114){
  sub_post[,i] <- as.numeric(sub_post[,i])
}

sub_post$K_SCORE <- rowSums(sub_post[, 105:114])
mean(sub_post$K_SCORE)
sd(sub_post$K_SCORE)

sub_post$K_SELF_mean <- rowSums(sub_post[, 56:61])/6
mean(sub_post$K_SELF_mean)
sd(sub_post$K_SELF_mean)


## attitude
sub_post$A7 = 6-sub_post$A7
sub_post$A8 = 6-sub_post$A8
sub_post$A9 = 6-sub_post$A9
sub_post$A10 = 6-sub_post$A10

sub_post$A_mean <- rowSums(sub_post[, 62:75])/14
mean(sub_post$A_mean)
sd(sub_post$A_mean)


sub_post_result <- c(mean(sub_post$K_SCORE),sd(sub_post$K_SCORE),
mean(sub_post$K_SELF_mean),sd(sub_post$K_SELF_mean) , mean(sub_post$A_mean),
sd(sub_post$A_mean))
result <- data.frame(un_pre_result, sub_pre_result, sub_post_result)
result <- t(result)

names <- c("K_mean", "K_sd", "K_self_mean", "K_self_sd", "A_mean", "A_sd")
colnames(result) <- names


################## un - post ######################

un_post <- read.csv(file = "Unsubscribers_已过滤.csv", header = TRUE)
back_up_un_post<-un_post


## calculate knowldege scores
```

```
for (i in 1:75) {
  if(!is.na(un_post$K1_1[i] == 1 | un_post$K1_2[i] == 2| un_post$K1_3[i] == 2|
un_post$K1_4[i] == 1| un_post$K1_5[i] == 1)){
    un_post$K1[i] <- "CORRECT"
  } else {
    un_post$K1[i] <- "WRONG"
  }
}

un_post_test <- un_post[,c("K1_1", "K1_2", "K1_3","K1_4","K1_5","K1")]

for (i in 1:75) {
  if(!is.na(un_post$K2_1[i] == 1 | un_post$K2_2[i] == 1| un_post$K2_3[i] == 2|
un_post$K2_4[i] == 1| un_post$K2_5[i] == 2)){
    un_post$K2[i] <- "CORRECT"
  } else {
    un_post$K2[i] <- "WRONG"
  }
}

for (i in 1:75) {
  if(!is.na(un_post$K3_1[i] == 1 | un_post$K3_2[i] == 1| un_post$K3_3[i] == 2|
un_post$K3_4[i] == 1| un_post$K3_5[i] == 2)){
    un_post$K3[i] <- "CORRECT"
  } else {
    un_post$K3[i] <- "WRONG"
  }
}

for (i in 1:75) {
  if(!is.na(un_post$K4_1[i] == 1 | un_post$K4_2[i] == 2| un_post$K4_3[i] == 2|
un_post$K4_4[i] == 1| un_post$K4_5[i] == 1)){
    un_post$K4[i] <- "CORRECT"
  } else {
    un_post$K4[i] <- "WRONG"
  }
}

for (i in 1:75) {
  if(!is.na(un_post$K5_1[i] == 1 | un_post$K5_2[i] == 1| un_post$K5_3[i] == 1|
un_post$K5_4[i] == 2| un_post$K5_5[i] == 1)){
    un_post$K5[i] <- "CORRECT"
  } else {
    un_post$K5[i] <- "WRONG"
  }
}
```

```
for (i in 1:75) {
  if(!is.na(un_post$K6_1[i] == 1 | un_post$K6_2[i] == 2| un_post$K6_3[i] == 1|
un_post$K6_4[i] == 2| un_post$K6_5[i] == 2)){
    un_post$K6[i] <- "CORRECT"
  } else {
    un_post$K6[i] <- "WRONG"
  }
}

for (i in 1:75) {
  if(!is.na(un_post$K7_1[i] == 2 | un_post$K7_2[i] == 2| un_post$K7_3[i] == 1|
un_post$K7_4[i] == 2| un_post$K7_5[i] == 2)){
    un_post$K7[i] <- "CORRECT"
  } else {
    un_post$K7[i] <- "WRONG"
  }
}

for (i in 1:75) {
  if(!is.na(un_post$K8_1[i] == 1 | un_post$K8_2[i] == 2| un_post$K8_3[i] == 2|
un_post$K8_4[i] == 2| un_post$K8_5[i] == 2)){
    un_post$K8[i] <- "CORRECT"
  } else {
    un_post$K8[i] <- "WRONG"
  }
}

for (i in 1:75) {
  if(!is.na(un_post$K9_1[i] == 2 | un_post$K9_2[i] == 1| un_post$K9_3[i] == 2|
un_post$K9_4[i] == 1| un_post$K9_5[i] == 2)){
    un_post$K9[i] <- "CORRECT"
  } else {
    un_post$K9[i] <- "WRONG"
  }
}

for (i in 1:75) {
  if(!is.na(un_post$K10_1[i] == 2 | un_post$K10_2[i] == 1| un_post$K10_3[i] == 1|
un_post$K10_4[i] == 1| un_post$K10_5[i] == 2)){
    un_post$K10[i] <- "CORRECT"
  } else {
    un_post$K10[i] <- "WRONG"
  }
}

un_post[un_post == "CORRECT"] <- 1
```

```
un_post[un_post == "WRONG"] <- 0

for (i in 87:96){
  un_post[,i] <- as.numeric(un_post[,i])
}

un_post$K_SCORE <- rowSums(un_post[, 87:96])
mean(un_post$K_SCORE)
sd(un_post$K_SCORE)

un_post$K_SELF_mean <- rowSums(un_post[, 56:61])/6
mean(un_post$K_SELF_mean)
sd(un_post$K_SELF_mean)


## attitude
un_post$A7 = 6-un_post$A7
un_post$A8 = 6-un_post$A8
un_post$A9 = 6-un_post$A9
un_post$A10 = 6-un_post$A10

un_post$A_mean <- rowSums(un_post[, 62:75])/14
mean(un_post$A_mean)
sd(un_post$A_mean)


## collate result
un_post_result <- c(mean(un_post$K_SCORE),sd(un_post$K_SCORE),
mean(un_post$K_SELF_mean),sd(un_post$K_SELF_mean) , mean(un_post$A_mean),
sd(un_post$A_mean))
result <- data.frame(un_pre_result, sub_pre_result, un_post_result, sub_post_result)
result <- t(result)
names <- c("K_mean", "K_sd", "K_self_mean", "K_self_sd", "A_mean", "A_sd")
colnames(result) <- names
```
