# Supplementary material for: Evaluating a WeChat-Based Intervention to Enhance Influenza Vaccination Knowledge, Attitude, and Behavior Among Chinese University Students Residing in the United Kingdom: Controlled, Quasi-Experimental, Mixed Methods Study
Source: JMIR Form Res. 2024 Oct 24;8:e55706. doi: 10.2196/55706 (PMC11544343; doi:10.2196/55706)
Supplement: Multimedia Appendix 3 [file formative_v8i1e55706_app3.docx]

Appendix 3. Statistics for between-group and within-group differences (corresponding to Figure 3)

|  | **Intervention (n = 303)** | | | **Control (n = 293)** | | | **Between group difference**  **p-value^a^** |
| --- | --- | --- | --- | --- | --- | --- | --- |
| Mean  (SE) | Before | After | Within-group difference^b^ | Before | After | Within-group difference^b^ |  |
| Knowledge | 5.756 (0.098) | 6.604  (0.102) | 0.845  (0.071), p<0.001 | 5.891 (0.109) | 5.901  (0.116) | 0.010 (0.048), p=0.831 | <0.001 |
| Attitude | 3.564 (0.026) | 3.762  (0.033) | 0.199  (0.041), p<0.001 | 3.688 (0.028) | 3.688  (0.028) | 0.002 (0.004), p=0.725 | <0.001 |
| Behavior(Intended) | 2.475 (0.049) | 2.883  (0.038) | 0.894  (0.058), p<0.001 | 2.451 (0.061) | 2.899  (0.054) | 0.877 (0.066), p<0.001 | 0.618 |
| Vaccination Rate, n (%) | - | 63 (20.8%) |  | - | 54  (18.4%) |  | - |
| 1. Differences in mean changes (follow up minus baseline) of KAB scores between intervention and control groups were conducted, p value calculated using clustered independent Wilcoxon Signed Rank tests. 2. *p*-value calculated using clustered independent Wilcoxon Signed Rank tests. | | | | | | | |
