# Supplementary material for: Evaluating a WeChat-Based Intervention to Enhance Influenza Vaccination Knowledge, Attitude, and Behavior Among Chinese University Students Residing in the United Kingdom: Controlled, Quasi-Experimental, Mixed Methods Study
Source: JMIR Form Res. 2024 Oct 24;8:e55706. doi: 10.2196/55706 (PMC11544343; doi:10.2196/55706)
Supplement: Multimedia Appendix 4 [file formative_v8i1e55706_app4.docx]

Appendix 4. Statistics for users’ feedback (corresponding to Figure 6)

| User’s feedback | Strongly agree | Agree | Neutral | Disagree | Strongly disagree |
| --- | --- | --- | --- | --- | --- |
| 1.I'm willing to continue following and using it | 121(0.399) | 118(0.389) | 49(0.162) | 15(0.05) | 0(0) |
| 2.It is easy to use. | 28(0.092) | 208(0.686) | 55(0.182) | 12(0.04) | 0(0) |
| 3.Its content and features are appealing. | 93(0.307) | 102(0.337) | 69(0.228) | 21(0.069) | 18(0.059) |
| 4.Its content and features align with my needs | 115(0.38) | 84(0.277) | 49(0.162) | 37(0.122) | 18(0.059) |
| 5.The contents are interesting. | 70(0.231) | 122(0.403) | 71(0.234) | 22(0.073) | 18(0.059) |
| 6.The information provided are trustworthy | 135(0.446) | 101(0.333) | 67(0.221) | 0(0) | 0(0) |
| 7.It is useful. | 94(0.31) | 105(0.347) | 49(0.162) | 37(0.122) | 18(0.059) |
| 8.I am willing to recommend it or its content  to my friends | 131(0.432) | 70(0.231) | 47(0.155) | 15(0.05) | 40(0.132) |
| 9.It is helpful in providing SIV information | 90(0.297) | 65(0.215) | 112(0.37) | 0(0) | 36(0.119) |
| 10.It is helpful in making SIV decisions | 82(0.271) | 58(0.191) | 148(0.488) | 0(0) | 15(0.05) |
